# Essential Role of the *a*3 Isoform of V-ATPase in Secretory Lysosome Trafficking via Rab7 Recruitment

**DOI:** 10.1038/s41598-018-24918-7

**Published:** 2018-04-30

**Authors:** Naomi Matsumoto, Mizuki Sekiya, Koujiro Tohyama, Eri Ishiyama-Matsuura, Ge-Hong Sun-Wada, Yoh Wada, Masamitsu Futai, Mayumi Nakanishi-Matsui

**Affiliations:** 1 Division of Biochemistry, School of Pharmacy, Iwate Medical University, Yahaba, Iwate 028-3694 Japan; 2 The Center for Electron Microscopy and Bio-Imaging Research, Iwate Medical University, Yahaba, Iwate 028-3694 Japan; 3 Department of Physiology, School of Dentistry, Iwate Medical University, Yahaba, Iwate 028-3694 Japan; 4 Department of Biochemistry, Faculty of Pharmaceutical Sciences, Doshisha Women’s College, Kyotanabe, Kyoto 610-0395 Japan; 5 Division of Biological Sciences, Institute of Scientific and Industrial Research, Osaka University, Ibaraki, Osaka 567-0047 Japan

## Abstract

Secretory lysosomes are required for the specialised functions of various types of differentiated cells. In osteoclasts, the lysosomal proton pump V-ATPase (vacuolar-type ATPase) is targeted to the plasma membrane via secretory lysosomes and subsequently acidifies the extracellular compartment, providing optimal conditions for bone resorption. However, little is known about the mechanism underlying this trafficking of secretory lysosomes. Here, we demonstrate that the lysosome-specific *a*3 isoform of the V-ATPase *a* subunit plays an indispensable role in secretory lysosome trafficking, together with Rab7, a small GTPase involved in organelle trafficking. In osteoclasts lacking *a*3, lysosomes were not transported to the cell periphery, and Rab7 was not localised to lysosomes but diffused throughout the cytoplasm. Expression of dominant-negative (GDP-bound form) Rab7 inhibited lysosome trafficking in wild-type cells. Furthermore, *a*3 directly interacted with the GDP-bound forms of Rab7 and Rab27A. These findings reveal a novel role for the proton pump V-ATPase in secretory lysosome trafficking and an unexpected mechanistic link with Rab GTPases.

## Introduction

Trafficking of organelles and membrane vesicles is indispensable for various types of cargo transport, including endocytosis and secretion. For example, bacterial toxins and low-density lipoprotein are internalised via endocytosis, transported to lysosomes, their final destination, and enzymatically digested in acidic conditions^1,2^. Lysosomes can also move in an anterograde direction and fuse with the plasma membrane, leading to secretion^3,4^. These so-called “secretory lysosomes” contain secretory proteins and hydrolysing enzymes^3,4^. Such lysosomes, which are observed in several cell types including platelets, spermatids and those involved in the immune system, are responsible for the specialised functions of various differentiated cells^3–6^. Melanocytes secrete melanin via lysosome-related organelles called melanosomes, leading to pigmentation^7^. Cytotoxic T lymphocytes and natural killer cells secrete a pore-forming protein via lysosomes to kill cells that need to be removed^8,9^. Defects of genes related to secretory lysosomes cause albinism and immunodeficiency in human and mouse^10,11^.

Secretory lysosomes are also indispensable in osteoclasts, which are involved in bone resorption^3,4,12^. These cells differentiate from progenitors upon stimulation with receptor activator of nuclear factor kappa B ligand (RANKL)^13^, attach to the bone surface via actin rings and form a resorption lacuna, an acidic compartment facing the bone surface^14–16^. Lysosomes with vacuolar-type ATPase (V-ATPase) in their membrane move to the periphery of osteoclasts and fuse with the plasma membrane^12,17,18^. Thereafter, lysosomal enzymes are secreted into the bone resorption lacuna^12,19,20^. Lysosomal V-ATPase that has relocalised to the plasma membrane acidifies the lacuna, providing optimal conditions for bone digestion and dissolution of calcium phosphate^17,18,21^.

V-ATPase, composed of catalytic V_1_ and proton pathway V_o_ sectors, transports protons across the membrane using energy obtained via ATP hydrolysis^22,23^. Six of the thirteen subunits that form mammalian V-ATPases have multiple isoforms specific to an organelle and/or type of differentiated cells^24–26^. Of the four *a* subunit isoforms forming a proton pathway in V_o_, *a*1, *a*2 and *a*3 are expressed ubiquitously, whereas *a*4 is expressed specifically in renal, reproductive and optic organs^23–26^. The *a*1, *a*2 and *a*3 isoforms are found in coated vesicles, early endosomes/Golgi apparatus and late endosomes/lysosomes, respectively^23–26^. Of the two *d* subunit isoforms connecting V_1_ and V_o_, *d*2 was recently shown to be osteoclast-specific^27–30^. Osteoclast V-ATPase mostly contains the *a*3 and *d*2 isoforms, and expression of both is significantly induced during differentiation^30^. Genetic defects of *a*3 increase bone density and thereby cause severe osteopetrosis in humans (OMIM 604592)^31^. In *a*3-deficient mice, osteoclasts fail to transport protons and exhibit impaired bone resorption^32,33^. Previous studies focused on *a*3 as an essential isoform of V-ATPase for acidification of lacunae.

Small GTPases belonging to the Rab family are key regulators of organelle trafficking^34,35^. More than 60 Rab proteins have been identified in mammalian cells^36,37^. Individual Rab proteins are recruited from the cytosol to distinct organelles or vesicles and connect them to the microtubule or actin cytoskeleton, in cooperation with specific effector proteins and other machinery^34,35,38^. The activities of Rab proteins are regulated by guanine nucleotides, with the GTP- and GDP-bound forms being active and inactive, respectively^34,35,38^. Reduced expression of Rab7 and Rab27A in osteoclasts results in impaired bone resorption, indicating that these proteins are involved in lysosomal secretion^39,40^. Despite recent intensive studies, the mechanism by which individual Rab proteins are recruited to specific organelles/vesicles remains to be elucidated.

Here, we demonstrate that the *a*3 isoform of V-ATPase has dual function in osteoclasts: it is essential not only for lacunae acidification, but also for secretory lysosome trafficking via Rab protein recruitment. We elucidate the mechanism underlying lysosome trafficking, demonstrating an unexpected link between the V-ATPase *a*3 isoform and Rab small GTPases.

## Results

### Localisation of lysosomal membrane proteins in osteoclasts from *a*3-knockout mice

**Figure 1:**
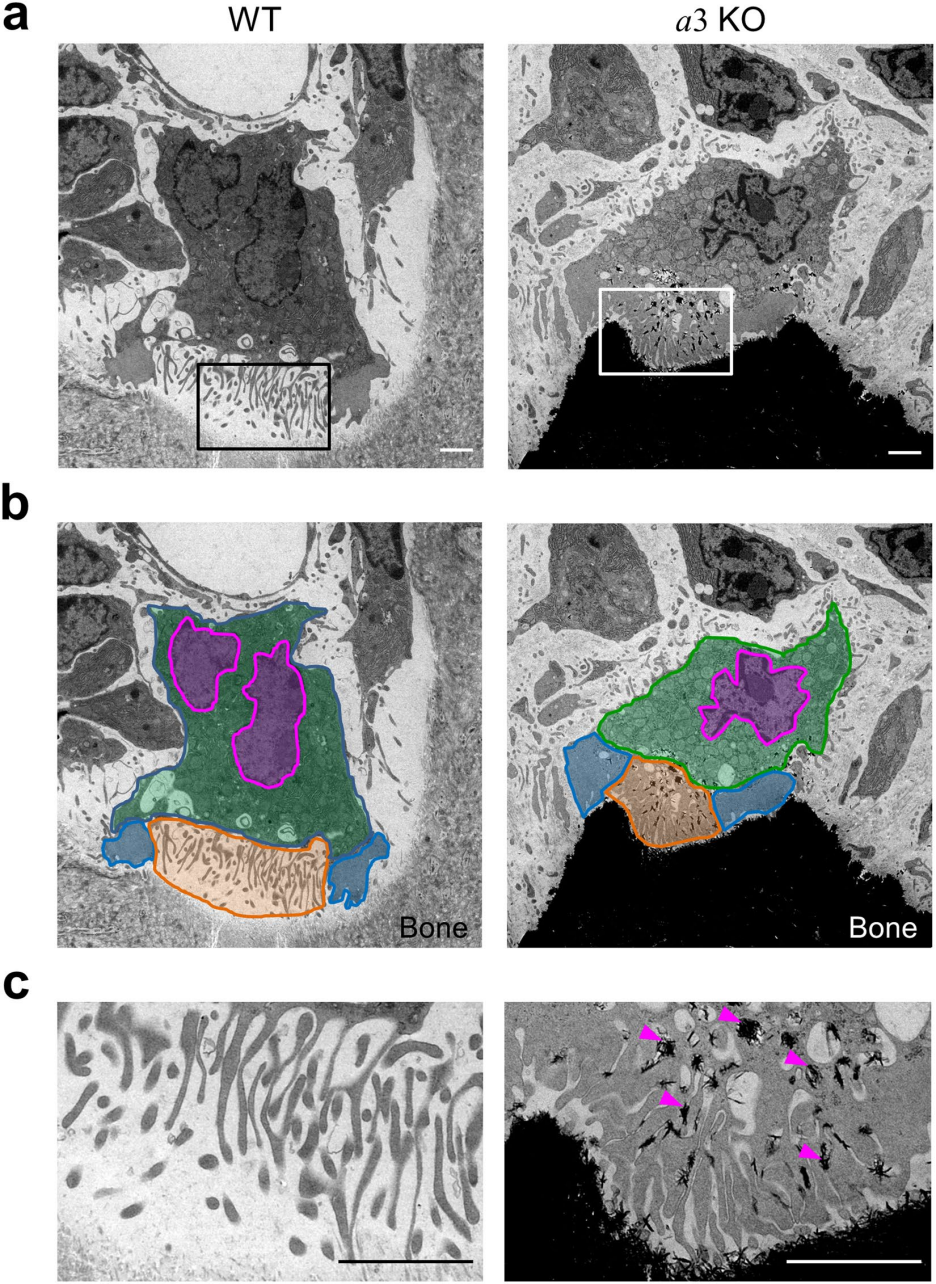
Osteoclasts in the epiphysis of humeral bones from wild-type and *a*3-knockout mice. Electron micrographs of a representative area containing osteoclasts in the humeral epiphysis from wild-type (**a**, WT) and *a*3-knockout (**a**, *a*3KO) mice are shown, together with coloured images (**b**). The ruffled border (orange), actin rings (blue), nucleus (magenta) and cytoplasm (green) are indicated schematically. An osteoclast faces the bone matrix with a finger-like ruffled border between the peripheral clear zone (actin ring). Higher magnification images of the boxed area in **a** are shown in **c**. In the mutant mouse, electron-dense material is found between processes of the ruffled border (**c**, arrowheads). The bone matrix is markedly more electron-opaque in the mutant mouse than in the wild-type mouse. The images are representative of 18 wild-type cells and 14 *a*3-knockout cells. Bars indicate 2 μm.

Osteoclasts, multinuclear cells that face the bone matrix, were observed in the humeral epiphysis of wild-type and *a*3-knockout mice by electron microscopy (Fig. 1a), confirming previous results^32,41^. These cells had peripheral clear zones (actin rings) and a characteristic ruffled border (Fig. 1b, blue and orange, respectively). We defined the cytoplasm of osteoclasts as all areas apart from the peripheral clear zones, nucleus and ruffled border (Fig. 1b, green). In higher magnification images, finger-like folds of the ruffled border were clearly identified (Fig. 1c). These results indicate that the *a*3 isoform is not essential for formation of lacunae. However, the bone matrix of mutant mice was electron-opaque compared with that of wild-type mice (Fig. 1a), suggesting that *a*3-deficient mice developed osteopetrosis. We also observed electron-dense material between processes of the ruffled border in mutant mice (Fig. 1c, arrowheads).

**Figure 2:**
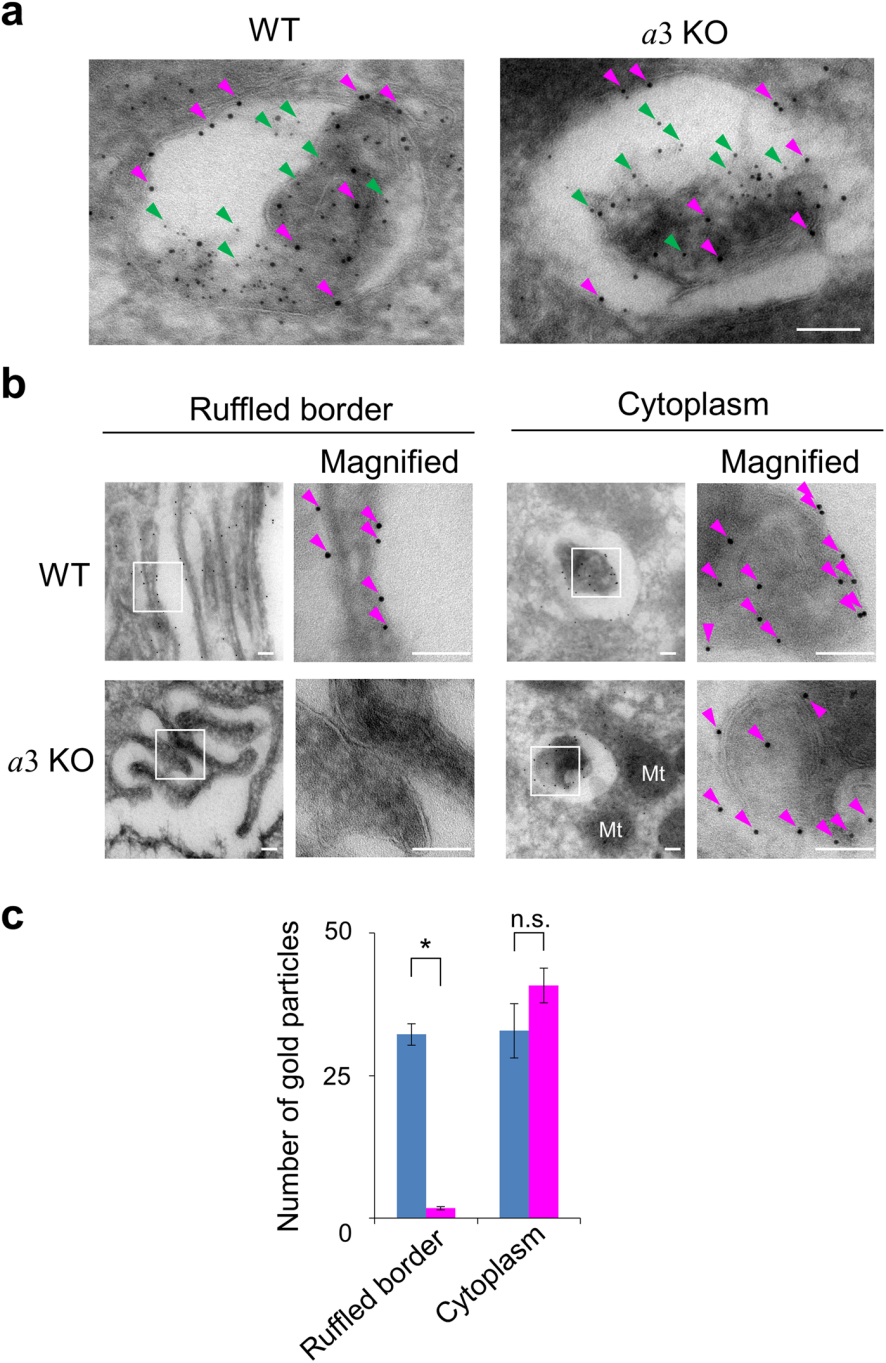
Localisation of CD68 in wild-type and *a*3-knockout osteoclasts. (**a**) Co-localisation of CD68 and cathepsin K in osteoclasts. Lysosomes in osteoclasts of humeri from wild-type (WT) and *a*3-knockout (*a*3KO) mice were visualised by indirect ultracryo-immunogold labelling. CD68, a late endosomal/lysosomal protein, and cathepsin K, a lysosomal enzyme, were labelled with 10 nm (examples, magenta arrowheads) and 5 nm (examples, green arrowheads) gold particles, respectively. The images are representative of ten cells. The bar indicates 100 nm. (**b**) Histochemical localisation of CD68 in wild-type (WT) and *a*3-knockout (*a*3KO) osteoclasts. Magnified images of the ruffled border and cytoplasm are shown. The boxed regions were further magnified for immunogold detection (Magnified). Arrowheads indicate 10 nm gold particles labelling CD68. The images are representative of ten cells. Bars indicate 100 nm. Particles were rarely observed in mitochondria and nuclei. (**c**) Intracellular distribution of CD68. The numbers of 10 nm gold particles were counted in 30 randomly selected fields (42 μm^2^) of the ruffled border and cytoplasm (*n = *1934 and 1278 particles for wild-type and mutant cells, respectively). One field measured 1.4 μm^2^, which corresponds to the area of panels without magnification in (**b**). Blue and magenta bars indicate wild-type and *a*3-knockout osteoclasts, respectively. The mean numbers of particles observed per field are shown together with s.e.m.; *p < 0.0001; n.s., not significant (unpaired two-tailed Student’s *t*-test). Only a few particles were found in the ruffled border of mutant osteoclasts.

Immunoelectron microscopy revealed that CD68 and cathepsin K, a trans-membrane lysosomal/late endosomal protein and a lysosomal protease, respectively, co-localised in organelles (100–450 nm diameter) in wild-type osteoclasts (Fig. 2a, magenta and green arrowheads, respectively). CD68 and cathepsin K also co-localised in *a*3-knockout cells (Fig. 2a). These results indicate that lysosomes in mutant and wild-type osteoclasts contained both cathepsin K and CD68.

Gold particles labelling CD68 were observed in the ruffled border membrane of wild-type osteoclasts (Fig. 2b, WT), consistent with the previous results indicating that secretory lysosomes fused with this membrane^21,42^. However, gold particles were rarely observed in the ruffled border of *a*3-knockout osteoclasts (Fig. 2b, *a*3KO). These particles were found in cytoplasmic organelles in both wild-type and *a*3-knockout osteoclasts (Fig. 2b, Cytoplasm).

The distribution of CD68 was determined by counting the number of gold particles in 30 randomly selected fields of the ruffled border and cytoplasm (Fig. 2c). There was a mean of 32.0 ± 2.0 and 2.0 ± 0.3 particles per field (1.4 μm^2^) in the ruffled border of wild-type and mutant osteoclasts, respectively. On the other hand, the number of gold particles in the cytoplasm was similar in wild-type and mutant osteoclasts. These results indicate that relocation of lysosomal CD68 to the cell surface is defective in *a*3-knockout osteoclasts.

### Lysosome trafficking in osteoclasts derived from mouse splenic macrophages

Trafficking of lysosomes to the plasma membrane was further studied in osteoclasts derived from splenic macrophages. Wild-type macrophages began to fuse after incubation with RANKL for 2 days and differentiated into multinuclear cells expressing the osteoclast marker tartrate-resistant acid phosphatase (TRAP) (Supplementary Fig. S1a, WT). Mutant macrophages differentiated into multinuclear osteoclasts with almost the same kinetics as wild-type macrophages (Supplementary Fig. S1a, *a*3KO). In addition, the numbers of TRAP-positive fused cells derived from mutant and wild-type macrophages were almost the same. However, mutant osteoclasts did not form any resorption pits when cultured on a calcium phosphate substrate mimicking bone^30^ (Supplementary Fig. S1b).

**Figure 3:**
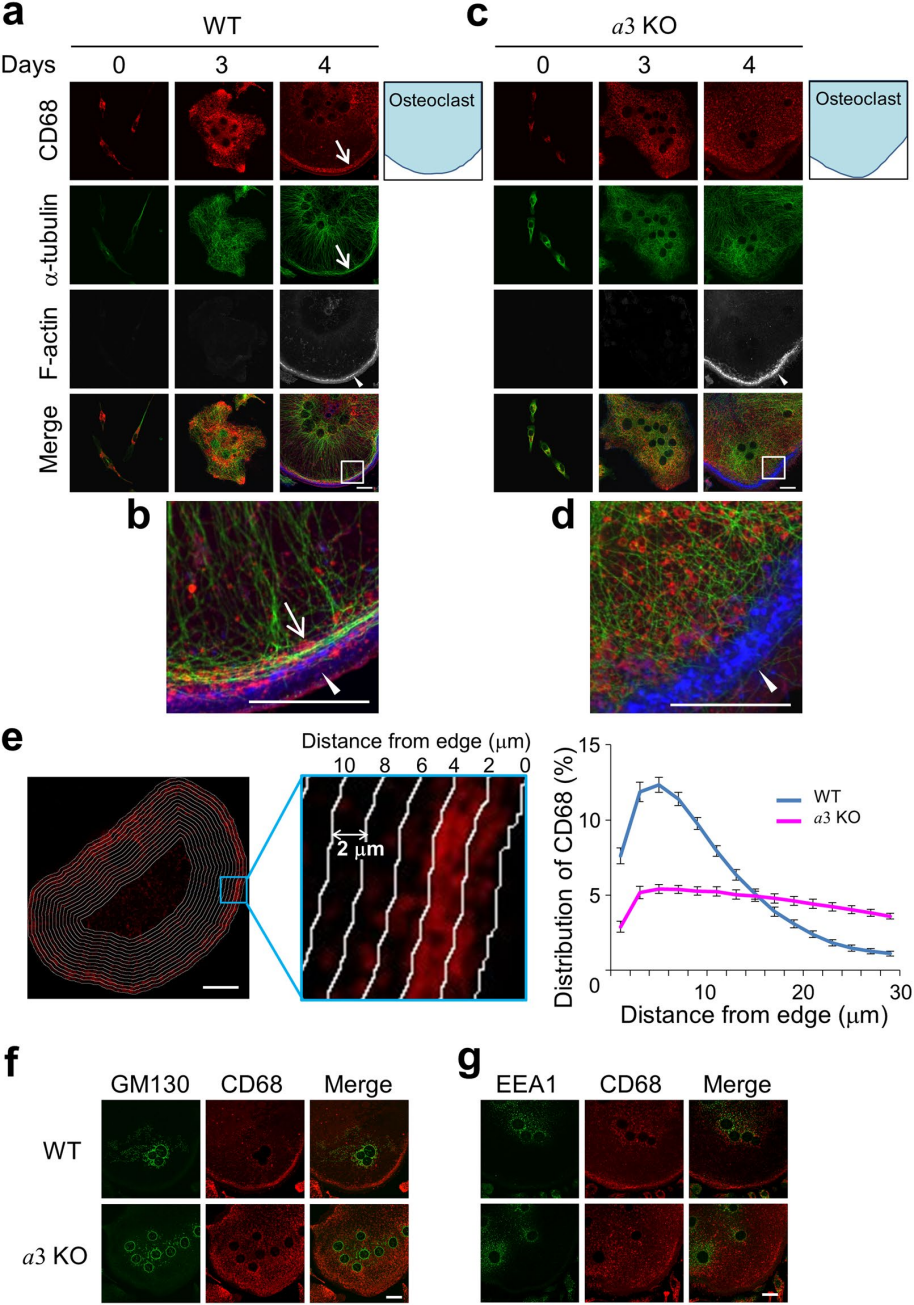
Localisations of organelles in osteoclasts differentiated from splenic macrophages. (**a**–**d**) Localisation of CD68 during differentiation. Macrophages from wild-type (**a**,**b**) and *a*3-knockout (**c**,**d**) mice were obtained as adherent splenic cells after incubation with macrophage colony-stimulating factor for 3 days, and then cultured in medium containing RANKL for 4 days to induce differentiation. The cells were then fixed and stained for CD68 (red) and α-tubulin (green). F-actin was visualised with phalloidin (white). F-actin labelling is shown in blue in the merged images. Merged images of CD68, α-tubulin and F-actin from day 4 were magnified (**b**,**d**). Schematic illustrations of osteoclasts (day 4) are also shown (blue). Arrows indicate CD68 and α-tubulin at the periphery of wild-type osteoclasts. Arrowheads indicate actin rings. The images are representative of at least 30 cells. Bars indicate 20 μm. (**e**) Distribution of CD68 in osteoclasts. An image of a cell differentiated as described in (**a**–**d**) was divided into 16 sections using the shape of the cell outline (left and middle panels). The width of each section was 2 μm. The intensity of CD68 staining in each section was quantified, and the relative intensity was plotted against the distance from the edge of the cell (right panel). Blue and magenta indicate wild-type and *a*3-knockout osteoclasts, respectively. Data are means ± s.e.m.; *n* = 15 cells. (**f**,**g**) Localisations of the Golgi (GM130) and early endosomes (EEA1) in osteoclasts. Splenic macrophages from wild-type (WT) and *a*3-knockout (*a*3KO) mice were cultured with RANKL for 4 days. Osteoclasts were fixed and stained with antibodies against GM130, a Golgi marker (**f**, green), EEA1, an early endosomal marker (**g**, green), and CD68 (red). Merged images of CD68 and GM130 or EEA1 are also shown (Merge). The images are representative of ten cells. Bars indicate 20 μm.

We then examined the localisations of the lysosomal protein CD68, α-tubulin and F-actin by immunostaining and phalloidin staining^17,30^. In wild-type cells, CD68 was observed mainly in the perinuclear area up to 3 days after RANKL addition (Fig. 3a, CD68). After 4 days, CD68-positive organelles were located close to the cell periphery (Fig. 3a, CD68, Days 4). These results suggest that lysosomes move to the cell periphery during differentiation. In addition, α-tubulin co-localised with CD68 close to the cell periphery, and an actin ring was observed adjacent to CD68 staining (Fig. 3a,b), consistent with our previous observation in osteoclasts differentiated from the murine macrophages line RAW264.7^17^. Unlike osteoclasts attached to bone, these cells were thin (~2 μm) and did not exhibit a highly developed ruffled border (Supplementary Fig. S1c).

In *a*3-knockout osteoclasts, CD68 and α-tubulin did not localise at the periphery (Fig. 3c,d), although the central microtubule network formed from tubulin and actin rings were observed (Fig. 3b,d). The central microtubule network appeared to be less radially oriented in mutant cells than in wild-type cells (Fig. 3b,d). Next, the distribution of CD68 was quantified by measuring its relative staining intensity in several sections created using the shape of the cell outline (Fig. 3e, left and middle). The relative intensity of CD68 staining was plotted against the distance from the cell edge (Fig. 3e, right). This revealed that 53% and 24% of CD68 staining was distributed within 10 μm from the edge of wild-type and *a*3-knockout osteoclasts, respectively. Similar results were obtained using another lysosome marker, LAMP1 (Supplementary Fig. S2).

On the other hand, GM130, a Golgi marker protein^43^, and EEA1, an early endosomal marker^44^, exhibited dot-like staining in the perinuclear region of both wild-type and *a*3-knockout cells (Fig. 3f,g). Taken together, these results indicate that the *a*3 isoform plays an essential role in outward trafficking of lysosomes and formation of the microtubule network at the cell periphery.

**Figure 4:**
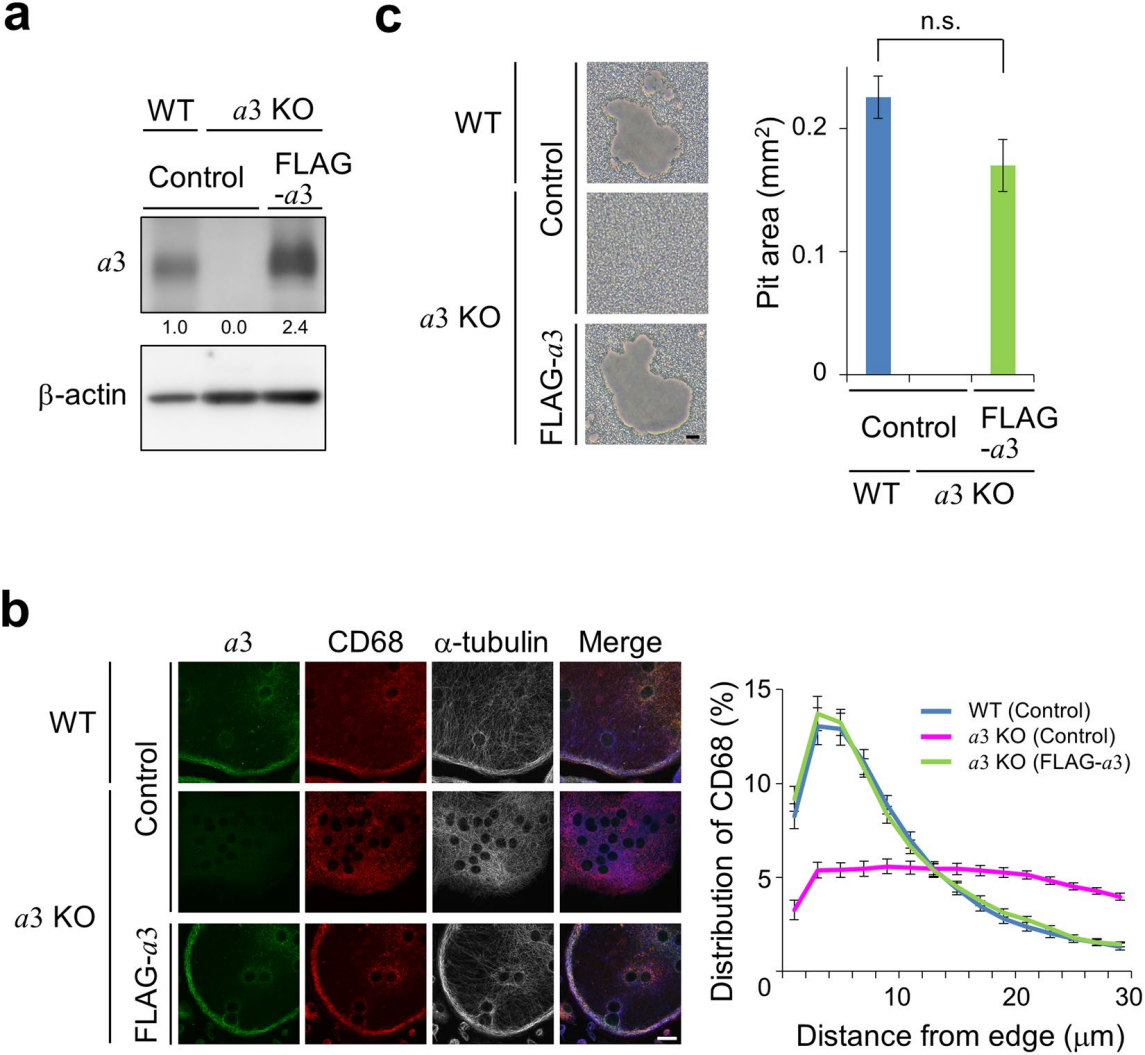
CD68 localisation and resorption activity of *a*3-knockout osteoclasts expressing FLAG-*a*3. (**a**) Expression of FLAG-*a*3 in *a*3-knockout osteoclasts. Splenic macrophages obtained from wild-type and mutant mice were transduced with pMX(puro) (empty vector) or pMX-FLAG-*a*3 (FLAG-*a*3), and cultured with RANKL for 6 days. Thereafter, osteoclast lysates were electrophoresed through an acrylamide gel. Endogenous and FLAG-*a*3 were detected with an antibody specific for *a*3 (upper panel). β-actin was also detected using corresponding antibody (lower panel). Numbers below blots indicate relative signal intensities of *a*3 normalised to that in wild-type cells. (**b**) Effects of *a*3 expression on the localisations of CD68 and α-tubulin in mutant osteoclasts. Osteoclasts induced as described in (**a**) were stained with antibodies specific for *a*3 (green), CD68 (red) and α-tubulin (white). Representative images are shown together with merged images (Merge). α-tubulin labelling is shown in blue in the merged images. The distribution of CD68 was determined (right panel) as described in Fig. 3e. Blue, magenta and green indicate wild-type, *a*3-knockout and FLAG-*a*3-expressing *a*3-knockout osteoclasts, respectively. Data are means ± s.e.m.; *n* = 15 cells. (**c**) Resorption activity of mutant osteoclasts expressing FLAG-*a*3. Osteoclasts were induced on calcium phosphate-coated dishes and then removed by washing with distilled water. Resorption pits were observed (left panels). The bar indicates 20 μm. Resorption activity was determined as the area of resorption pits in a field measuring 1.6 mm^2^ (right panel). Data are means ± s.e.m.; n.s., not significant (unpaired two-tailed Student’s *t*-test); *n* = 9 views.

When mutant macrophages expressing FLAG-*a*3 were differentiated into osteoclasts, lysosome localisation at the cell periphery was restored to the same level as that in wild-type cells (Fig. 4a,b). Moreover, calcium phosphate resorption activity of mutant osteoclasts expressing FLAG-*a*3 was restored to 76% of that of wild-type cells (Fig. 4c). These results confirm that *a*3 is required for localisation of lysosomes at the cell periphery.

Protein levels of the *a*1 and *a*2 isoforms in mutant osteoclasts were the same as those in wild-type osteoclasts (Supplementary Fig. S3). This indicates that their expression does not increase to compensate for the lack of *a*3 and that these isoforms cannot perform the role of *a*3 in lysosome trafficking.

We investigated the localisation of CD68 in mouse osteoclasts attached to the bone surface (Fig. 2) and in those differentiated from macrophages *in vitro* (Fig. 3). In both cases, the peripheral localisation of lysosomes required *a*3, and lysosomes were transported from the perinuclear region to the cell periphery.

### Effects of *a*3 on cytokine secretion and endocytosis

**Figure 5:**
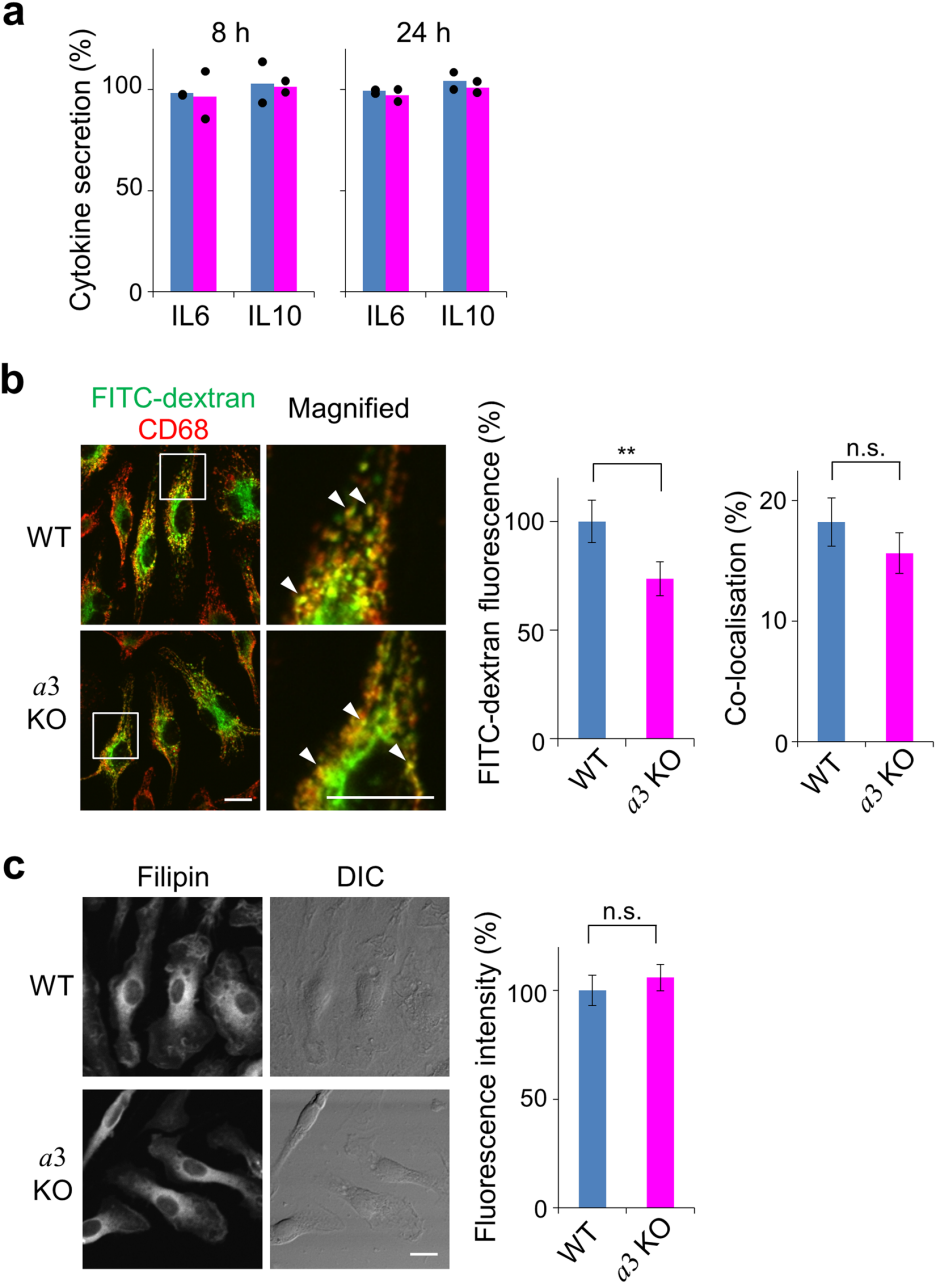
Cytokine secretion, endocytosis and cholesterol distribution in splenic macrophages. (**a**) IL6 and IL10 secretion by macrophages. Splenic macrophages from wild-type (blue) and *a*3-knockout (magenta) mice were cultured with lipopolysaccharide for 8 or 24 h, and the levels of IL6 and IL10 were determined by ELISAs. Secretion was quantitated as the amount of cytokine secreted into the medium against the total amount of cytokine synthesised. The bars show the means of technical duplicates. The dots indicate independent values. The levels of synthesised cytokines differed by about 2-fold between wild-type and mutant macrophages. (**b**) Endocytosis of dextran in macrophages. Splenic macrophages from wild-type (WT) and *a*3-knockout (*a*3KO) mice were incubated with FITC-dextran (green) for 30 min, washed and immunostained with an antibody against CD68 (red). FITC-dextran and CD68 were co-localised (Magnified, arrowheads). Bars indicate 10 μm. The relative fluorescence intensities of FITC-dextran are shown, with that in wild-type cells set to 100% (middle panel). Data are means ± s.e.m.; **p < 0.05 (unpaired two-tailed Student’s *t*-test); *n* = 131 wild-type and 170 mutant cells. Co-localisation of FITC-dextran and CD68 was determined as the percentage of FITC-positive pixels that were also CD68-positive (right panel). Data are means ± s.e.m.; n.s., not significant (unpaired two-tailed Student’s *t*-test); *n* = 30 cells. (**c**) Cholesterol distribution. Splenic macrophages from wild-type (WT) and *a*3-knockout (*a*3KO) mice were fixed, incubated with filipin for 30 min and then washed. DIC indicates differential interference contrast. The bar indicates 10 μm. The fluorescence intensity of filipin was assayed. Relative intensities are shown, with that in wild-type cells set to 100% (right panel). Data are means ± s.e.m.; n.s., not significant (unpaired two-tailed Student’s *t*-test); *n* = 94 wild-type cells and 108 mutant cells.

We investigated the effects of *a*3 knockout on other types of trafficking, namely, cytokine secretion^45^, endocytosis of FITC-dextran^46^ and cholesterol localisation^47^. To analyse cytokine secretion, macrophages from mutant and wild-type mice were incubated with lipopolysaccharide (LPS) for 8 or 24 h, and then the amounts of interleukin 6 (IL6) and interleukin 10 (IL10) secreted via Golgi-derived vesicles were assayed^48^. Secretion was quantitated as the amount of cytokine secreted into the medium against the total amount of cytokine synthesised. The percentage secretion of IL6 and IL10 by wild-type macrophages was about 100% (Fig. 5a, blue), indicating that almost all synthesised cytokine molecules were secreted. The same finding was made in mutant macrophages (Fig. 5a, magenta), indicating that *a*3 has no significant role in trafficking during cytokine secretion.

Next, we examined the effect of *a*3 knockout on endocytosis. FITC-dextran was taken up from the medium and partly localised in CD68-positive lysosomes in both wild-type and mutant macrophages (Fig. 5b, left panels). The amount of FITC-dextran taken up by mutant cells was about 75% of that taken up by wild-type cells (Fig. 5b, middle panel). Co-localisation of FITC-dextran and CD68 was also determined to assess delivery of FITC-dextran to late endosomes/lysosomes. About 18% and 16% of FITC-dextran co-localised with CD68 in wild-type and mutant cells, respectively (Fig. 5b, right panel). Although *a*3 knockout slightly reduced dextran uptake, these results indicate that *a*3 does not play an essential role in vesicle trafficking from the cell surface to late endosomes/lysosomes.

In many lysosomal storage disease (LSD) cells, accumulation of cholesterol in lysosomes promotes inward trafficking of lysosomes^47^. Filipin staining of cholesterol is significantly intense in the perinuclear region of these cells^47^. Filipin staining in the perinuclear region was similar in *a*3-knockout and wild-type cells (Fig. 5c, left panels), suggesting that cholesterol localisation in mutant cells is similar to that in wild-type cells. Additionally, in contrast with LSD cells, the level of cholesterol was not higher in *a*3-knockout cells than in wild-type cells (Fig. 5c, right panel). These results indicate that *a*3 has no role in the localisation or accumulation of cholesterol. Thus, the defective lysosome trafficking observed in mutant osteoclasts is not due to altered cholesterol accumulation in the perinuclear region.

### Involvement of Rab small GTPases in lysosome trafficking

Rab GTPases regulate organelle trafficking and positioning by linking organelles/vesicles to cytoskeletal motor proteins^34,35^. Rab7 functions in transport from late endosomes to lysosomes^49^. Rab27A (a Rab27 isoform) is involved in secretion of lysosome-related organelles^50,51^. Reduced expression of these two proteins results in impaired bone metabolism^39,40^. Furthermore, secretory lysosomes are formed in lymphocytes via the merger of late endosomes and recycling endosomes containing Rab7/Rab27A and Rab11, respectively^51,52^. Of the two isoforms of Rab11, Rab11B is expressed in osteoclasts^53^.

**Figure 6:**
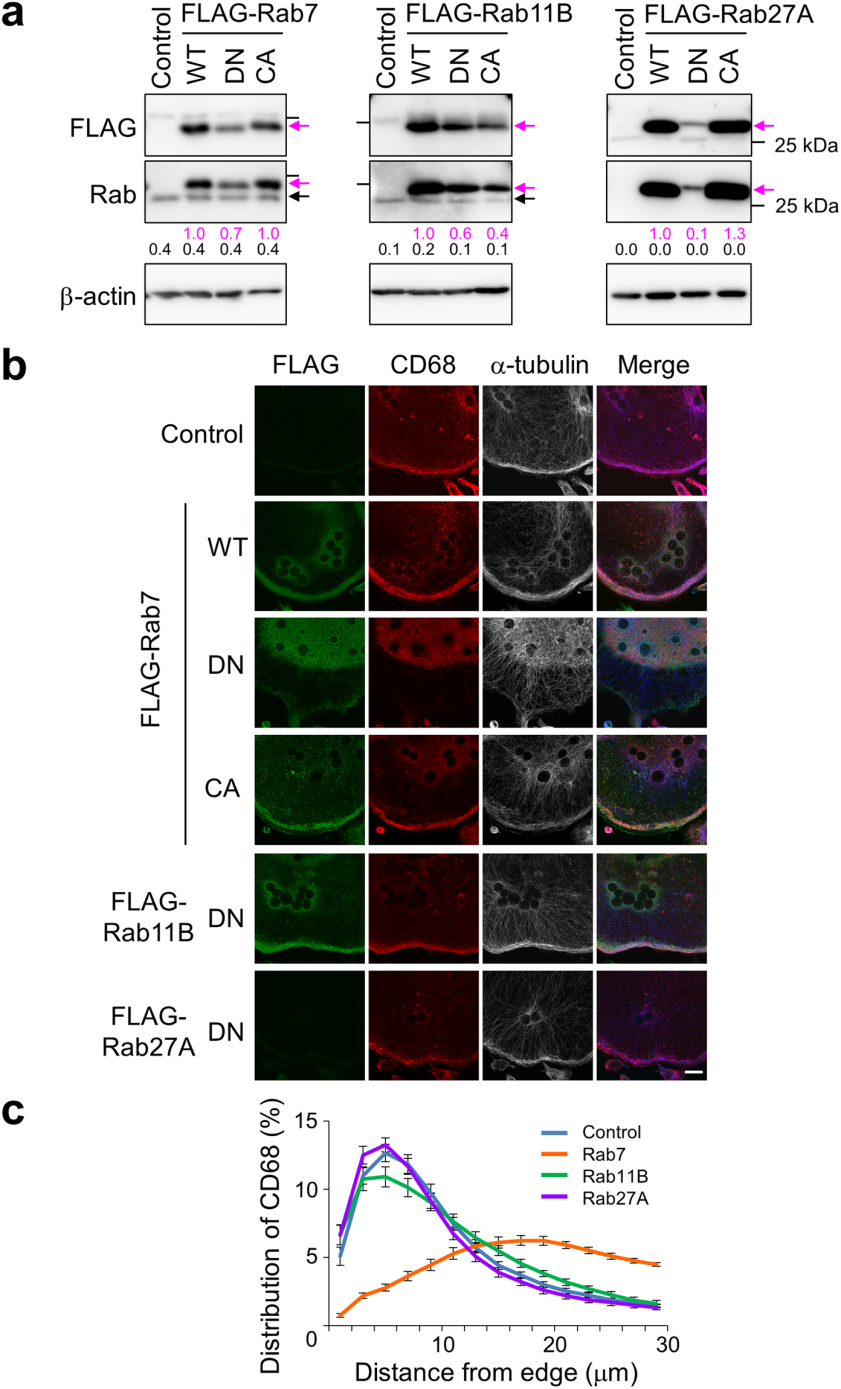
Effects of Rab small GTPases on the peripheral localisation of CD68. (**a**) Expression of various Rab proteins in osteoclasts. Splenic macrophages from wild-type mice were infected with a retrovirus carrying a gene encoding FLAG-tagged Rab7 (left panels), Rab11B (middle panels) or Rab27A (right panels) and cultured with RANKL for 6 days. Then, osteoclast lysates were electrophoresed through an acrylamide gel. FLAG-Rab proteins were detected with antibodies specific for FLAG (upper panel, magenta arrows) and each Rab protein (middle panel, magenta arrows). Endogenous Rab proteins (middle panel, closed arrows) and β-actin (lower panel) were also detected using corresponding antibodies. WT, DN and CA indicate cells expressing wild-type, dominant-negative (GDP-bound) and constitutively active (GTP-bound) Rab proteins, respectively. Control indicates cells infected with an empty vector. Bars indicate the position of the 25 kDa molecular mass marker. Numbers below blots represent relative signal intensities of FLAG-tagged (magenta) and endogenous (black) Rab protein normalised to that of FLAG-tagged wild-type Rab protein. Unprocessed scans of immunoblots are shown in Supplementary Fig. S6. (**b**) Effects of Rab protein expression on the peripheral localisation of CD68. Osteoclasts expressing Rab proteins were prepared as described in (**a**) and stained with antibodies against FLAG (green), CD68 (red) and α-tubulin (white). Merged images (Merge) are also shown. α-tubulin labelling is shown in blue in the merged images. WT, DN, CA and Control are as described in (**a**). The images are representative of at least nine cells. The bar indicates 20 μm. (**c**) Distribution of CD68 in osteoclasts expressing dominant-negative Rab proteins. Osteoclasts were stained as described in (**b**). The intensity of CD68 staining in each section was quantified and summarised as described in Fig. 3e. Blue, orange, green and purple indicate control and dominant-negative Rab7-, Rab11B- and Rab27A-expressing osteoclasts, respectively. Data are means ± s.e.m.; *n* = 15 cells.

To examine the roles of these Rab GTPases, we overexpressed their FLAG-tagged dominant-negative (GDP-bound), wild-type and constitutively active (GTP-bound) forms in wild-type macrophages (Fig. 6, DN, WT and CA, respectively), and then induced differentiation into osteoclasts. FLAG-tagged Rab proteins were detected by immunoblotting (Fig. 6a, upper panels, magenta arrows). FLAG-tagged and endogenous Rab proteins were simultaneously detected using specific antibodies against each Rab protein (Fig. 6a, middle panels, magenta and closed arrows, respectively). Signals of FLAG-tagged Rab proteins were markedly stronger than those of endogenous Rab proteins.

FLAG-Rab7, CD68 and α-tubulin were localised at the periphery of osteoclasts derived from macrophages expressing wild-type or constitutively active FLAG-Rab7, but were localised in the perinuclear region of osteoclasts derived from macrophages expressing the dominant-negative form (Fig. 6b, FLAG-Rab7, DN and 6c). These results indicate that dominant-negative Rab7 suppresses outward lysosome trafficking. Overexpressed dominant-negative Rab proteins are often dispersed throughout the cytoplasm^54,55^; however, in this study, dominant-negative Rab7 was localised at the perinuclear region with lysosomes in osteoclasts. Our results also indicate that dominant-negative Rab7 suppressed formation of the peripheral microtubule network (Fig. 6b, FLAG-Rab7, α-tubulin). Activated Rab7 can interact with α-tubulin^56^. Therefore, the peripheral localisation of lysosomes may be required for tethering of microtubules at the cell periphery and, conversely, the peripheral microtubule network may promote accumulation of secretory lysosomes near to the plasma membrane. The same results were obtained when the various forms of Rab7 were expressed after differentiation (Supplementary Fig. S4).

The dominant-negative forms of Rab11B and Rab27A did not affect the localisations of CD68 and α-tubulin (Fig. 6b, FLAG-Rab11B and FLAG-Rab27A, and 6c). The wild-type and constitutively active forms of these Rab proteins also had no effect (Supplementary Fig. S5). These results suggest that Rab11B and Rab27A are not involved in lysosome trafficking in osteoclasts. Therefore, we analysed Rab7 in comparison with Rab11 or Rab27 in subsequent experiments.

### Lysosomal localisation of Rab7

**Figure 7:**
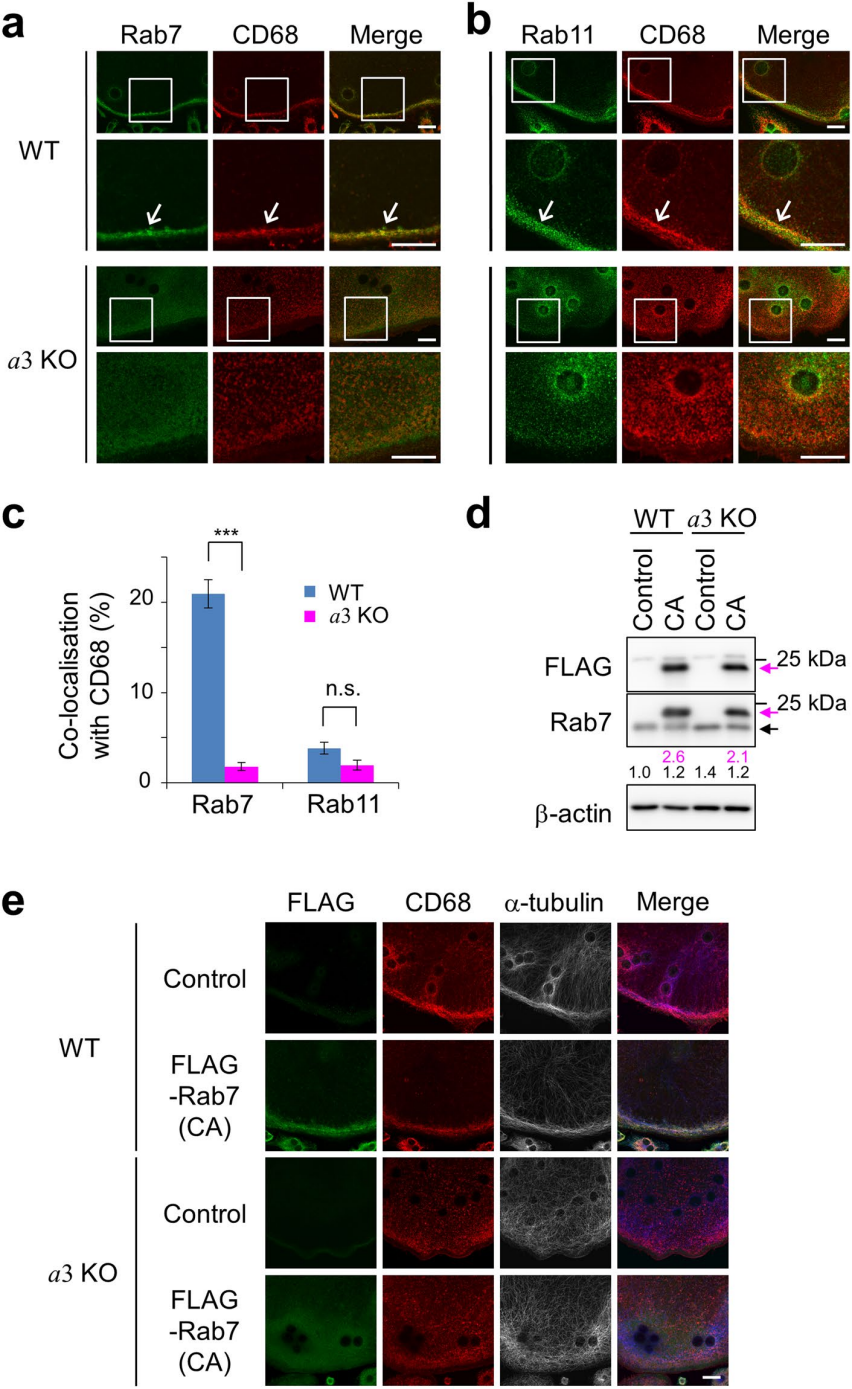
Localisations of Rab proteins and CD68 in osteoclasts. (**a**,**b**) Localisations of CD68, Rab7 and Rab11 in osteoclasts. Osteoclasts derived from wild-type (WT) and *a*3-knockout (*a*3KO) macrophages were stained with antibodies against CD68 (red) and Rab7 (**a**, green) or Rab11 (**b**, green). Representative images are shown, together with merged images (Merge). The boxed areas are magnified in the lower panels. The images are representative of at least ten cells. Bars indicate 20 μm. (**c**) Co-localisation of CD68 and Rab7 or Rab11. Co-localisation is quantified as the percentage of CD68-positive pixels that were also Rab protein-positive. Blue and magenta bars indicate osteoclasts derived from wild-type and *a*3-knockout macrophages, respectively. At least five osteoclasts were examined per experiment. Data are means ± s.e.m.; ***p < 0.0005; n.s., not significant (unpaired two-tailed Student’s *t*-test). (**d**) Expression of constitutively active FLAG-Rab7. Wild-type (WT) and *a*3-knockout (*a*3KO) macrophages were infected with a retrovirus expressing constitutively active FLAG-Rab7 and then cultured in the presence of RANKL for 6 days to induce osteoclast differentiation. Lysates of these osteoclasts were prepared and analysed by SDS-polyacrylamide gel electrophoresis and Western blotting using the indicated antibodies. Magenta and closed arrows indicate FLAG-Rab7 and endogenous Rab7, respectively. Control and CA indicate cells infected with an empty vector and those expressing constitutively active Rab7, respectively. Bars indicate the position of the 25 kDa molecular mass marker. Numbers below blots represent relative signal intensities of FLAG-tagged (magenta) and endogenous (black) Rab7 normalised to that of endogenous Rab7 in wild-type cells. Unprocessed scans of immunoblots are shown in Supplementary Fig. S6. (**e**) Localisation of constitutively active FLAG-Rab7. Wild-type (WT) and *a*3-knockout (*a*3KO) osteoclasts expressing constitutively active FLAG-Rab7 were prepared as described in (**d**) and stained with antibodies against FLAG (green), CD68 (red) and α-tubulin (white). Merged images (Merge) are also shown. α-tubulin labelling is shown in blue in the merged images. Control and CA are as described in (**d**). The images are representative of at least eight cells. Bars indicate 20 μm.

We examined the localisation of Rab7 in wild-type and mutant osteoclasts. Rab7 co-localised with CD68 at the periphery of wild-type osteoclasts (Fig. 7a, WT). On the other hand, Rab7 was diffusely located throughout the cytoplasm in *a*3-knockout osteoclasts and did not co-localise with CD68 (Fig. 7a, *a*3KO). CD68 was diffused throughout mutant osteoclasts; however, unlike Rab7, it exhibited a clear dot-like staining pattern (Fig. 7a, *a*3KO). These results suggest that Rab7 fails to associate with CD68-positive compartments upon loss of *a*3, and thus that *a*3 is involved in the recruitment of Rab7 to lysosomes.

We examined the localisation of Rab11, a marker of recycling endosomes^52^, for comparison. In wild-type osteoclasts, Rab11 co-localised with CD68 only at the cell periphery (Fig. 7b, WT), suggesting that secretory lysosomes are formed via the merger of lysosomes and recycling endosomes in osteoclasts, similar to lymphocytes^51^. In *a*3-knockout cells, Rab11 and CD68 did not co-localise at the cell periphery (Fig. 7b, *a*3KO). Recycling endosomes can move to the plasma membrane in a lysosome-independent manner^57^; however, our results indicate that their peripheral localisation is dependent on lysosome trafficking, at least in osteoclasts. Unlike Rab7, Rab11 had a dot-like staining pattern in mutant osteoclasts (Fig. 7b, *a*3KO), confirming that *a*3 is not required for recruitment of Rab11 to recycling endosomes. To quantitatively compare the co-localisation of CD68 and Rab proteins, we determined the percentage of CD68-positive pixels that were also Rab protein-positive in the merged images. Co-localisation of CD68 with Rab7 was significantly lower in mutant cells (~2%) than in wild-type cells (~20%) (Fig. 7c, Rab7). On the other hand, CD68 hardly co-localised with Rab11 in both mutant (~2%) and wild-type (~4%) cells (Fig. 7c, Rab11). These results indicate that *a*3 promotes the lysosomal localisation of Rab7.

GTP-bound Rab proteins localise in organelle/vesicle membranes^35^. Therefore, we tested whether *a*3 is required for lysosomal localisation of constitutively active (GTP-bound) Rab7. In both wild-type and mutant osteoclasts, constitutively active FLAG-Rab7 was expressed at a higher level than the endogenous protein (Fig. 7d, magenta and closed arrows, respectively). In wild-type cells, it localised at the cell periphery together with CD68 and α-tubulin (Fig. 7e, WT). By contrast, in *a*3-knockout cells, constitutively active FLAG-Rab7 was dispersed throughout the cytoplasm and did not localise to a specific organelle, and the peripheral localisation of CD68 and α-tubulin was not recovered (Fig. 7e, *a*3KO). These results indicate that *a*3 is essential for the localisation of GTP-bound Rab7 to secretory lysosomes.

### Association of *a*3 with Rab GTPases

**Figure 8:**
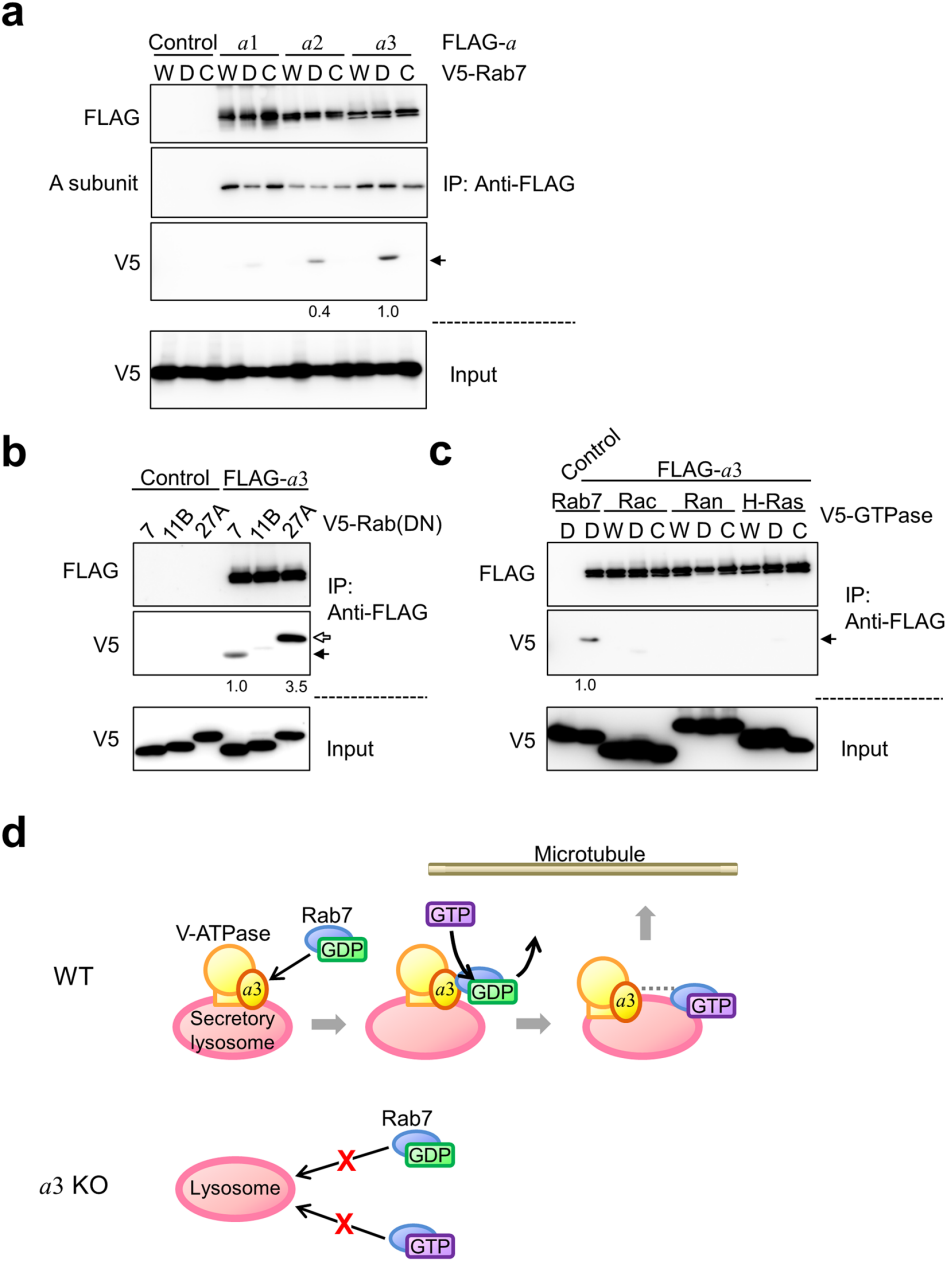
Interaction of *a* subunit isoforms with small GTP-binding proteins. (**a**) Interaction of *a* subunit isoforms and Rab7. FLAG-tagged *a* isoforms and various V5-fused forms of Rab7 were co-expressed in HEK293T cells. The cells were lysed, and lysates were immunoprecipitated with an anti-FLAG antibody. The precipitates were analysed using antibodies against FLAG (upper panel), the A subunit of the V_1_ sector (upper middle panel) and V5 (lower middle panel). As a control, cells were co-transfected with an empty vector and a recombinant plasmid harbouring V5-fused Rab7 (Control). W, D and C indicate wild-type, dominant-negative GDP-bound (T22N) and constitutively active GTP-bound (Q67L) Rab7, respectively. About 5% of the cell lysate used for immunoprecipitation was also subjected to Western blotting with an anti-V5 antibody (lower panel). Dominant-negative Rab7 co-precipitated with FLAG-*a*3 (middle panel, arrow). Numbers below blots represent relative signal intensities of V5-Rab7 co-precipitated with *a* isoform normalised to that co-precipitated with *a*3. Relative intensities less than 0.1 are not shown. (**b**) Interaction of *a*3 and dominant-negative Rab proteins. FLAG-*a*3 and the V5-fused dominant-negative form of Rab7, Rab11B or Rab27A were co-expressed in HEK293T cells. Immunoprecipitation was carried out as described in (**a**). Rab7 and Rab27A were detected in the precipitate (middle panel, closed and open arrows, respectively). Numbers below blots represent relative signal intensities of V5-fused Rab protein co-precipitated with *a*3 normalised to that of V5-Rab7. Relative intensities less than 0.1 are not shown. (**c**) Interaction of *a*3 and the Rac, Ran or H-Ras GTP-binding protein. FLAG-*a*3 and V5-fused Rac, Ran or H-Ras were co-expressed in HEK293T cells. Immunoprecipitation was carried out as described in (**a**). Numbers below blots represent relative signal intensities of V5-fused GTP-binding protein co-precipitated with *a*3 normalised to that of V5-Rab7. Relative intensities less than 0.1 are not shown. Unprocessed scans of immunoblots in (**a**–**c**) are shown in Supplementary Fig. S6. (**d**) Model illustrating the role of *a*3 in secretory lysosome trafficking. During osteoclast differentiation, the *a*3 isoform, the level of which is increased, recruits GDP-bound Rab7, and GDP is subsequently exchanged for GTP via an unknown GEF. Activated Rab7 anchors secretory lysosomes to microtubules, in collaboration with other machineries that are omitted here. Interestingly, *a*3 is also required for the localisation of GTP-bound Rab7 to lysosomes. The dotted line between *a*3 and GTP-bound Rab7 indicates a functional interaction between these two proteins.

*a*3 was required for the lysosomal localisation of Rab7 (Fig. 7); therefore, we investigated whether these two proteins directly interact. We co-expressed a FLAG-tagged *a* subunit isoform (*a*1, *a*2 or *a*3) and a V5-fused Rab7 variant (wild-type, dominant-negative or constitutively active) in HEK293T cells, and performed immunoprecipitations with an anti-FLAG antibody. The catalytic A subunit of V-ATPase was detected in the precipitate (Fig. 8a, A subunit). Given that the A and *a* subunits are in V_1_ and V_o_, respectively, this result suggests that the FLAG-tagged *a* subunit and other subunits assembled to form V-ATPase. V5-fused dominant-negative Rab7, but not wild-type or constitutively active Rab7, co-precipitated with FLAG-*a*3, whereas it was not as efficiently precipitated with the *a*1 or *a*2 isoform (Fig. 8a, arrow). These results indicate that dominant-negative Rab7 specifically associates with *a*3.

We also examined the association of *a*3 with the dominant-negative forms of Rab11B and Rab27A. Rab27A was immunoprecipitated with *a*3, whereas no obvious precipitation of Rab11B was observed (Fig. 8b). To further examine the binding specificity, we selected other small GTP-binding proteins from different subfamilies. Although Rac, Ran and H-Ras were expressed at a similar level as Rab7, they were not immunoprecipitated with *a*3 (Fig. 8c).

Unexpectedly, we detected an association between *a*3 and Rab27A. This prompted us to examine whether *a*3 is required for lysosomal localisation of this Rab protein. FLAG-Rab27A localised diffusively in mutant osteoclasts, whereas it co-localised with CD68 at the periphery of wild-type cells (Supplementary Fig. S4b,c). This suggests that *a*3 is also involved in the recruitment of Rab27A to lysosomes.

Taken together, *a*3 interacts specifically with the dominant-negative forms of Rab7 and Rab27A. Given that these forms mimic GDP-bound Rab proteins, GDP-bound Rab7 may be recruited to lysosomes via a direct interaction with *a*3, and, after nucleotide exchange, the activated Rab proteins may associate with microtubules for trafficking to the cell periphery (Fig. 8d).

## Discussion

This study revealed an unexpected mechanistic link between the *a*3 isoform of V-ATPase and Rab7 in osteoclasts. Therefore, *a*3 has dual functions in osteoclasts; it is not only an essential isoform of the proton pump, but is also involved in secretory lysosome trafficking.

Rab7 localised to lysosomes in an *a*3-dependent manner, and its dominant-negative (GDP-bound) form specifically interacted with *a*3. These results suggest that *a*3 recruits GDP-bound Rab7 from the cytosol to secretory lysosomes. After its activation, GTP-bound Rab7 associates with microtubules to mediate trafficking (Fig. 8d, WT). Lysosomes lacking *a*3 did not recruit GDP-bound Rab7 and hence were not transported to the cell periphery (Fig. 8d, *a*3KO). Unexpectedly, *a*3 was also required for the lysosomal localisation of activated Rab7 (Fig. 8d, *a*3KO).

Levels of lysosome-specific *a*3, which is a component of the V_o_ sector, a ubiquitous B2 isoform, which is a component of the V_1_ sector, and the *d*2 isoform, which connects the V_o_ and V_1_ sectors, increase during differentiation of progenitors into osteoclasts^30^. As described above, the catalytic A subunit of V_1_ was immunoprecipitated with *a*3. These results suggest that *a*3 forms V-ATPase together with other subunits in secretory lysosomes and recruits a sufficient amount of Rab7 to tightly anchor lysosomes to microtubules for long-range delivery to the cell periphery.

Rab7 regulates late endosome/lysosome trafficking in both outward and inward directions. Several factors are involved in trafficking together with Rab7. Rab-interacting lysosomal protein (RILP) collaborates with Rab7 as an adaptor for the motor protein dynein, which moves in an inward direction along microtubules^56^. FYVE and coiled-coil domain-containing 1 (FYCO1) is an adaptor for the motor protein kinesin, which moves in an outward direction^56^. Thus, FYCO1 is hypothesised to function in trafficking of secretory lysosomes.

Guanine nucleotide exchange factors (GEFs) are other essential proteins for organelle transport regulated by small GTPases. These factors bind to the corresponding GDP-bound Rab protein and activate it by exchanging GDP for GTP^34,35,58^. Therefore, the complex containing *a*3 and Rab7 likely has GEF activity for Rab7. Although this complex may include the factors discussed above, they were omitted from the model for simplicity (Fig. 8d). The Mon1-Ccz1 complex is the GEF for Rab7^59^. However, a recent study concerning on macropinocytosis induced in Cos-7 cells demonstrated that Mon1-Ccz1 is recruited to late endosomes but subsequently detaches from lysosomes^60^. This previous study also indicates that Ccz1 depletion does not affect lysosome positioning that requires Rab7 activation^60^. These findings suggest that Mon1-Ccz1 is not involved in activation of lysosomal Rab7. Further studies are required to fully elucidate the lysosome trafficking mechanism, including the roles of the Rab7 GEF and its interaction with *a*3.

The specific interaction between *a*3 and GDP-bound Rab27A (Fig. 8) and the *a*3-dependent lysosomal localisation of Rab27A (Supplementary Fig. S5b,c) suggest that *a*3 also recruits this protein to the lysosomal membrane. Recent studies of lysosomal secretion in cytotoxic T lymphocytes revealed that Rab27A is involved in docking and fusion between secretory vesicles and the plasma membrane^51^. More recently, Rab27A was suggested to play a role in the transport of cell surface receptors in osteoclasts^40^. Taken together, *a*3 is likely involved in docking and fusion between secretory lysosomes and the plasma membrane via recruitment of Rab27A.

Secretion via lysosomes and related organelles is observed in various types of cells, such as cytotoxic T lymphocytes, melanocytes, platelets and spermatids^3–6^. *a*3 is a ubiquitously expressed lysosome-specific isoform^17,61^. Therefore, *a*3 is likely involved in trafficking of secretory lysosomes in these cells. In addition, insulin secretion by β cells in Langerhans islets is decreased in *a*3-knockout mice^62^. *a*3 is found in insulin secretory granules and other endocrine tissues^62,63^, although they are not lysosome-related organelles. Therefore, the mechanisms underlying regulated secretion may be similar to that underlying lysosomal secretion in osteoclasts. On the other hand, V-ATPase in lysosome-related organelle is suggested to be involved in inward trafficking in HeLa cells based on the finding that loss of its function promotes secretion of multivesicular bodies in the cells^64^. Thus, V-ATPase plays a role in both outward and inward trafficking of lysosomes and lysosome-related organelles. This is consistent with the fact that Rab7 regulates both outward and inward trafficking. The direction of the trafficking appears to be dependent on the cell types.

*a*3 knockout did not affect the localisations of early endosomes and the Golgi in osteoclasts. The *a*1 and *a*2 isoforms localise in coated vesicles and early endosomes/Golgi, respectively^24–26^. Neither isoform could perform the role of *a*3 in lysosome trafficking in osteoclasts. These results indicate that the three isoforms have independent roles in organelle trafficking and positioning. In this regard, previous studies reported the role of the *a*1 and *a*2 isoforms in intracellular trafficking pathways. In *Drosophila* central neurons, *a*1 has an essential role in secretion of neurotransmitter from synaptic vesicles^65^. In mouse kidney proximal cells, *a*2 in early endosomes is involved in vesicle trafficking from early to late endosomes during endocytosis of macromolecules^66^. *a*2 recruits ADP-ribosylation factor nucleotide site operator (ARNO), a GEF for the small GTPase Arf6, which facilitate vesicle trafficking from early to late endosomes^66,67^.

Altogether, organelle-specific *a* subunit isoforms play an important role in determining the direction of organelle trafficking by recruiting specific co-factors including small GTPases. Further studies of the roles of V-ATPase isoforms will establish the mechanism underlying organelle trafficking.

## Methods

### Animals and cell culture

Wild-type and *a*3-knockout mice were generated as described previously^30,68^. Briefly, C57BL/6-*a*3^+/−^ mice (BRC no. 04421) were obtained from RIKEN BioResource Center and crossed to generate C57BL/6-*a*3^+/+^ (wild-type) and C57BL/6-*a*3^−/−^ (*a*3-knockout) mice. *a*3-knockout mice lack exons 15–20 of the *a*3 gene^68^. Macrophages were obtained as adherent cells after incubation of splenic cells from 2-week-old mice (male and female) in Minimum Essential Medium alpha (MEMα) supplemented with 10% foetal bovine serum, 100 U/mL penicillin, 100 μg/mL streptomycin and 25 ng/mL macrophage colony-stimulating factor (R&D Systems) for 3 days^30^. To induce osteoclast differentiation, splenic macrophages were cultured in the same medium containing 200 ng/mL RANKL (Peprotech) for 4 days^30^. Animals were used under the Guidelines for the Animal Experiments of Iwate Medical University and the Act on Welfare and Management of Animals of Japan. Animal protocols were approved by the Ethics Committee for Animal Research of Iwate Medical University (approval number: 28-017). HEK293T and Plat-E cells were purchased from RIKEN BioResource Center (RCB2202) and Cell Biolab, respectively. These cells were cultured in Dulbecco’s Modified Eagle Medium containing 10% foetal bovine serum, 100 U/mL penicillin and 100 μg/mL streptomycin. Unless otherwise indicated, all reagents used for cell culture were from Life Technologies. For the pit formation assay, osteoclasts were cultured on calcium phosphate-coated dishes (CORNING)^30^.

### Antibodies

Antibody information can be found in Supplementary Table S1. Antibodies against CD68 (FA-11), Rab27A and V5 were purchased from Hycult Biotechnology (HM1070), Proteintech (17817-1-AP) and Life Technologies (R96025), respectively. Antibodies to GM130 (618022) and LAMP1 (553792) were purchased from BD Biosciences Pharmingen. Antibodies against α-tubulin (DM1A, T9026), β-actin (AC-15, A5441) and FLAG (F7425) were from SIGMA. Anti-Rab7 (D95F2, 9367) and anti-Rab11 (D4F5, 5589) were obtained from Cell Signaling. Antibodies against cathepsin K (E-7, sc-48353) and GFP (B-2, sc-9996) were from Santa Cruz. Antibodies against *a*1, *a*2 and *a*3 were generated as described previously^30,61,69^. Alexa-conjugated secondary antibodies (A11034, A11029, A11081 and A21236) and colloidal gold-conjugated ones (EMGAT10 and EMGMHL5) were from Life Technologies and BBI solutions, respectively. HRP-conjugated antibodies to rabbit IgG (NA934VS), mouse IgG (NA931VS), chicken IgY (12–341) and native primary antibodies (21230) were purchased from GE healthcare (anti-rabbit IgG and mouse IgG), Millipore and Thermo Scientific.

### Electron microscopy

Two-week-old wild-type and *a*3-knockout mice were anesthetised and perfused with phosphate-buffered saline (137 mM NaCl, 27 mM KCl, 81 mM Na_2_HPO_4_ and 14.7 mM KH_2_PO_4_, pH 7.4) containing 4% paraformaldehyde. Humeri were isolated and soaked in 0.1 M phosphate buffer containing 2% paraformaldehyde and 2.5% glutaraldehyde overnight for ordinary electron microscopy or 4% paraformaldehyde for 4 h at 4 °C for immunogold electron microscopy. Fixed specimens were immersed in EDTA solution (9% EDTA-2Na-2H_2_0 and 10% EDTA-4Na-4H_2_O) at 4 °C for 1 week^17^.

For ordinary electron microscopy, osmification was carried out in 1% (w/v) osmium tetroxide solution, and decalcified bone tissues were dehydrated using a graded ethanol series and embedded in Epon812 (TAAB Laboratories). Ultrathin sections were cut using an ultramicrotome, stained with lead citrate plus uranyl acetate and then examined with a transmission electron microscope (H-7650, Hitachi)^70^.

Immunogold electron microscopy was carried out as described previously^71^. Briefly, samples were infused with 0.1 M phosphate buffer (pH 7.0) containing 20% polyvinylpyrrolidone and 1.8 M sucrose for 2 h at room temperature, incubated overnight at 4 °C and frozen at −190 °C using rapid-freezing apparatus (KF-80, Leica). Ultrathin cryosections were cut on an ultramicrotome (UCT or UC6, Leica) equipped with a cryoattachment (FCS or FC6, Leica). The sections were mounted on carbon-coated grids and incubated with primary antibodies (rat anti-CD68 (1:100) and mouse anti-cathepsin K (1:100)) at 4 °C for 48 h. After washing, the sections were incubated with a colloidal gold (10 nm)-conjugated anti-rat IgG antibody (BBI, 1:100) and/or a gold (5 nm)-conjugated anti-mouse IgG antibody (BBI, 1:100) at room temperature for 2 h. The grids were coated with poly(vinyl alcohol) containing 0.1% uranyl acetate and observed with an electron microscope (H-7650, Hitachi).

### Fluorescence microscopy

Immunostaining was performed as described^17^. Cells were fixed with 4% paraformaldehyde for 30 min and permeabilised in phosphate-buffered saline containing 0.4% saponin, 1% bovine serum albumin and 2% normal goat serum at room temperature for 15 min. Cells were incubated with primary antibodies at 4 °C overnight and then with fluorescent dye-conjugated secondary antibodies (Life Technologies) at room temperature for 1 h. F-actin was visualised with Alexa647-conjugated phalloidin (Life Technologies)^17,30^. Endocytosis and cholesterol localisation were examined using FITC-dextran (Life Technologies)^46^ and filipin (SIGMA)^47^, respectively. Fluorescence images were acquired with FV-1000 Confocal Microscope (OLYMPUS).

### ELISAs

Macrophages obtained from wild-type and *a*3-knockout mice were incubated with 100 ng/mL highly purified LPS (TLR grade, Alexis Biochemicals) for 8 or 24 h. After treatment, the culture media were collected and cell lysates were prepared^45^. ELISAs for IL6 and IL10 were performed as described in the manufacturer’s protocol (Thermo Scientific). Cytokine secretion (%) was defined as the amount in the culture medium relative to the total amount in the culture medium and cell lysate.

### Construction of plasmids

To construct a recombinant plasmid for expression of mouse *a*3, a DNA fragment encoding FLAG-tagged *a*3 was PCR-amplified using the pKT-mouse *a*3 plasmid^66^ as a template and primers *a*3_FW and *a*3_RV (Supplementary Table S2), and cloned into pcDNA3.1 using *Kpn*I and *Not*I. Recombinant plasmids for expression of FLAG-*a*1 and FLAG-*a*2 were generated by replacing the coding region of *a*3 in pcDNA3.1 with that of *a*1 or *a*2 in pKT^69^. To construct the V5-tagged Rab7 expression plasmid, a DNA fragment encoding Rab7 was obtained by reverse transcriptase-polymerase chain reaction (RT-PCR) using total RNA isolated from RAW264.7 cells and primers with a *Bam*HI or *Not*I site (Supplementary Table S2), and digested with *Bam*HI and *Not*I. The fragment and a V5-tag linker with *Kpn*I and *BamH*I sites were subcloned into pcDNA3.1 using *Kpn*I and *Not*I. Expression plasmids for other mouse small GTPases (Rab11B, Rab27A, Rac1, Ran and H-Ras) were created by replacing the Rab7 coding region with the DNA fragment encoding each gene, which was amplified by RT-PCR using the respective primers (Supplementary Table S2).

For retrovirus infection, FLAG-*a*3 and FLAG-Rab7, FLAG-Rab11B and FLAG-Rab27A were subcloned from pcDNA3.1 into the pMX(puro) plasmid by ligating the fragments digested with *Bam*HI and *Not*I. Dominant-negative and constitutively active mutations of small GTPases were introduced into the recombinant plasmids by oligonucleotide-directed site-specific mutagenesis PCR (Supplementary Table S2). EGFP-tagged Rab7 and mutant cDNAs were subcloned into the pAd/PL-DESTTM Gateway Vector for adenovirus construction (Invitrogen). The wild-type, dominant-negative and constitutively active forms of Rab7 were fused to the carboxyl terminal of GFP under the control of the cytomegalovirus promoter.

### Transfection and virus infection

HEK293T and Plat-E cells were transfected using ExtremeGENE9 (SIGMA) as described in the manufacturer’s protocol. Splenic macrophages were infected with retroviruses using a Platinum Retrovirus Expression System (Cell Biolab). Infected cells were selected in MEMα containing 2 μg/mL puromycin for 2 days, cultured for a further 2 days in the absence of the antibiotic and then incubated with 200 ng/mL RANKL for 6 days. Infected macrophages were incubated with RANKL for longer than non-infected macrophages because osteoclast differentiation occurred more slowly after retrovirus infection and selection with puromycin. Recombinant adenoviruses were constructed using the ViraPower Adenoviral Gateway Expression Kit (Invitrogen). The obtained adenoviruses were used to express EGFP-Rab7 variants in osteoclasts induced from macrophages.

### Immunoprecipitation assay and Western blotting

HEK293T cells were transfected with plasmids encoding a FLAG-tagged subunit *a* isoform and a V5-tagged small GTPase, lysed in IP buffer (1% Triton X-100, 10% glycerol, 50 mM Tris-HCl pH 7.4, 150 mM NaCl, 1 mM dithiothreitol, 1 mM EDTA, 1 mM phenylmethanesulfonyl fluoride and protease inhibitor cocktails) and immunoprecipitated with an anti-FLAG antibody as described previously^30,72^. Immunoprecipitates were analysed by Western blotting using Clean Blot (Thermo Scientific) as a secondary antibody. HRP-conjugated host-specific secondary antibodies (GE Healthcare) were used for Western blotting of macrophage and osteoclast lysates. Immune complexes were detected by chemiluminescence using an ECL prime detection kit (GE Healthcare) and an LAS-3000 imaging system (FUJIFILM).

### Quantitative analysis

To analyse the distribution of CD68 in electron microscopy images, the number of colloidal gold particles was counted in 30 randomly selected fields (1.4 μm^2^/field) of each area (ruffled border and cytoplasm). Ten randomly selected cells were analysed. In total, at least 1200 gold particles were counted in both wild-type and mutant osteoclasts.

To quantify the distribution of CD68 in confocal microscopy images, an image of a differentiated cell was divided into 16 sections using the shape of the cell outline. The width of each section was 2 μm. Thereafter, the fluorescence intensity of CD68 staining in each section was measured using Image-J software (NIH)^73^.

The signal fluorescence intensities (FITC-dextran and filipin), area of bone resorption pits and signal intensity of Western blotting were also quantified using Image-J. Cells fixed before addition of FITC-dextran were used as a negative control in the analysis of endocytosis. To determine the intracellular background labelling of filipin, the fluorescence intensities in three randomly selected areas (0.96 μm^2^/area) near to the plasma membrane were averaged as described previously^47^. Co-localisation of CD68 with Rab proteins or FITC-dextran was examined using a confocal FV-1000 microscope^74,75^.

### Statistics and reproducibility

The F-test and unpaired two-tailed Student’s *t*-test were performed using Microsoft Excel software for statistical comparisons. p < 0.05 was considered statistically significant. When representative images are shown, the numbers of samples examined are all indicated in the figure legends. All replications were successful, provided that progenitors differentiated into osteoclasts.

### Data availability

Source data for Figs 2c, 3e, 4b, 5a–c, 6c, 7c, S1a–b and S2b has been provided in Supplementary Table S3. All other data supporting the findings of this study are available from the authors on reasonable request.

## Electronic supplementary material

Supplementary Information

Supplementary Table S1

Supplementary Table S2

Supplementary Table S3
