# Supplementary material for: Essential Role of the a3 Isoform of V-ATPase in Secretory Lysosome Trafficking via Rab7 Recruitment
Source: Sci Rep. 2018 Apr 30;8:6701. doi: 10.1038/s41598-018-24918-7 (PMC5928161; doi:10.1038/s41598-018-24918-7)
Supplement: Supplementary file 1 — Supplementary Information [file 41598_2018_24918_MOESM1_ESM.pdf]

Essential Role of the *a*3 Isoform of V-ATPase in Secretory Lysosome Trafficking via Rab7 Recruitment

Naomi Matsumoto, Mizuki Sekiya, Koujiro Tohyama, Eri Ishiyama-Matsuura, Ge-Hong Sun-Wada, Yoh Wada, Masamitsu Futai, Mayumi Nakanishi-Matsui

Supplementary Figure S1. Differentiation of osteoclasts from splenic macrophages.
(**a**) Differentiation of macrophages into osteoclasts. Macrophages from wild-type (WT) and *a*3-knockout (*a*3KO) mice were obtained as adherent splenic cells after incubation with macrophage colony-stimulating factor for 3 days, cultured in medium containing RANKL for 4 days to induce differentiation and then stained for TRAP. The images are representative of at least nine fields. The bar indicates 50 μm. The numbers of TRAP-positive fused cells were counted in nine randomly selected fields (65.7 mm^2). Data are means ± s.e.m.; n.s., not significant (unpaired two-tailed Student's t-test). (**b**) Resorption activity of wild-type (WT) and *a*3-knockout (*a*3KO) osteoclasts. Osteoclasts were induced as described in **a** on calcium phosphate-coated dishes and then removed by washing with distilled water. Resorption pits were observed. The images are representative of nine fields. The bar indicates 20 μm. Resorption activity was determined by measuring the area of resorption pits in nine randomly selected fields (10.6 mm^2). Data are means ± s.e.m. (**c**) Images of osteoclasts induced from wild-type (WT) and *a*3-knockout (*a*3KO) macrophages. CD68 (red) and α-tubulin (green) were stained with specific antibodies. F-actin (blue) was stained with phalloidin. Lateral images (z-x and z-y sections along the yellow lines) are also shown. The image is representative of nine cells. The bar indicates 20 μm.

Supplementary Figure S2. Localisations of LAMP1 in osteoclasts differentiated from splenic macrophages.
(**a**) Localisation of LAMP1 in osteoclasts. Osteoclasts were derived from wild-type (WT) and *a*3-knockout (*a*3KO) macrophages as described in Figure 3**a**. The cells were then fixed and stained for LAMP1 (red) and α-tubulin (green). F-actin was visualised with phalloidin (white). F-actin labelling is shown in blue in the merged images. Arrows indicate LAMP1 and α-tubulin at the periphery of wild-type osteoclasts. Arrowheads indicate actin rings. The images are representative of at least ten cells. Bars indicate 20 μm. (**b**) Distribution of LAMP1 in osteoclasts. The distribution of LAMP1 in wild-type and mutant osteoclasts was determined as described in Figure 3**e**. Blue and magenta indicate osteoclasts derived from wild-type and *a*3-knockout macrophages, respectively. Data are means ± s.e.m.; n = 15 cells.

Supplementary Figure S3. Expression of the *a*1 and *a*2 isoforms during differentiation.
Osteoclasts were induced from wild-type (WT) and *a*3-knockout (*a*3KO) splenic macrophages. Lysates were prepared after the indicated number of days and subjected to Western blotting using antibodies specific for the *a*1 and *a*2 isoforms, and β-actin. Numbers below blots represent relative signal intensities of *a* isoform normalised to that at day 0.Unprocessed scans of immunoblots are shown in Supplementary Figure S6.

Supplementary Figure S4. Effects of Rab protein expression on the peripheral localisation of CD68 after differentiation.
(**a**) Expression of EGFP-Rab7 variants in osteoclasts. Wild-type osteoclasts induced from macrophages were infected with an adenovirus carrying a gene encoding EGFP-Rab7 and cultured for a further 24 h.

Cell lysates were subjected to gel electrophoresis. EGFP-Rab7 was detected with antibodies specific for GFP (upper panel, magenta arrow) and Rab7 (lower panel, magenta arrow). Endogenously expressed Rab7 and β-actin were also detected using the corresponding antibodies (lower panel, closed arrow; upper panel, blue arrow, respectively). WT, DN and CA indicate cells expressing wild-type, dominant-negative (GDP-bound) and constitutively active (GTP-bound) Rab7, respectively. Control indicates non-infected cells. Arrowheads indicate the positions of the 50, 37 and 25 kDa molecular mass markers. Numbers below blots represent relative signal intensities of EGFP-fused (magenta) and endogenous (black) Rab7 normalised to that of EGFP-fused wild-type Rab7. Unprocessed scans of immunoblots are shown in Supplementary Figure S6. (**b**) Effects of EGFP-Rab7 variants on the peripheral localisation of CD68. Osteoclasts expressing EGFP-Rab7 variants were prepared as described in **a** and stained with antibodies against CD68 (red) and α-tubulin (white). EGFP-Rab7 variants were detected by observing EGFP via fluorescence microscopy (green). Merged images (Merge) are also shown. α-tubulin labelling is shown in blue in the merged images. WT, DN, CA and Control are as described in **a**. The images are representative of at least three cells. The bar indicates 20 μm.

Supplementary Figure S5. Effects of Rab11B and Rab27A expression on the peripheral localisation of CD68.

(**a–c**) Splenic macrophages from wild-type mice were infected with a retrovirus carrying a gene encoding a FLAG-tagged Rab11B (**a**) or Rab27A (**b**) variant (wild-type, dominant-negative or constitutively active). In addition, macrophages from *a*3-knockout mice were infected with a retrovirus carrying a gene encoding FLAG-tagged wild-type Rab27A (**c**). Thereafter, infected cells were cultured with RANKL for 6 days. Osteoclasts expressing Rab protein were stained with antibodies against FLAG (green), CD68 (red) and α-tubulin (white). Merged images (Merge) are also shown. α-tubulin labelling is shown in blue in the merged images. WT, DN and CA indicate cells expressing wild-type, dominant-negative (GDP-bound) and constitutively active (GTP-bound) Rab proteins, respectively. Control indicates cells infected with an empty vector. The boxed images are the same with those shown in Figure 5**b** (FLAG-Rab11B DN and FLAG-Rab27A DN). The images are representative of at least nine cells. The bar indicates 20 μm.

Supplementary Figure S6. Unprocessed scans of immunoblots.

Supplementary Table Legends.

Supplementary Table S1. Information on antibodies used for this study.

Supplementary Table S2. Information on primers used for RT-PCR.

Supplementary Table S3. Statistics source data.

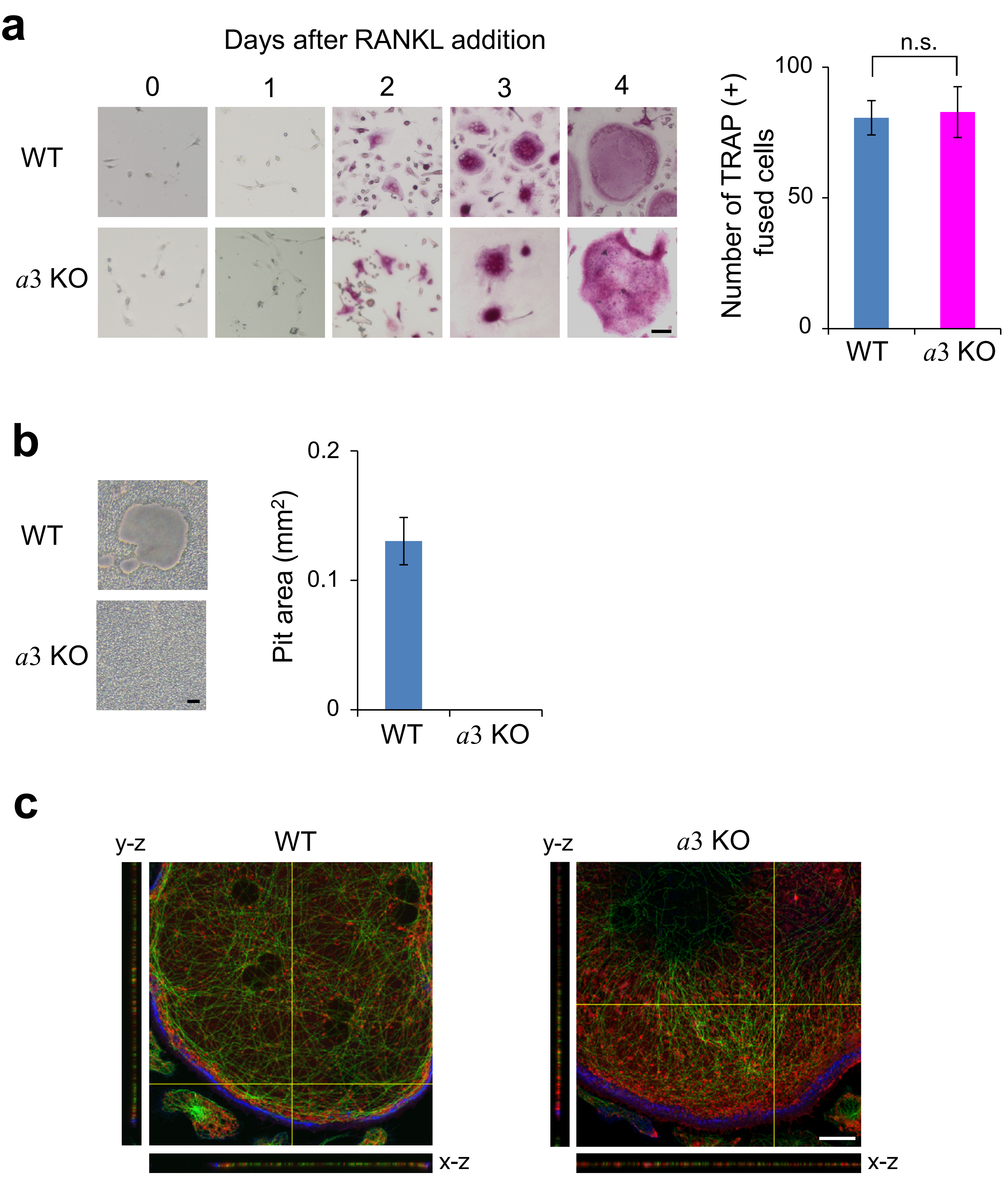

Supplementary Figure S1

a

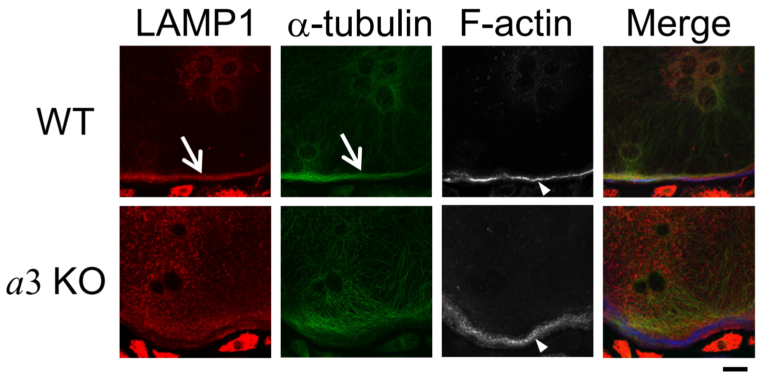

b

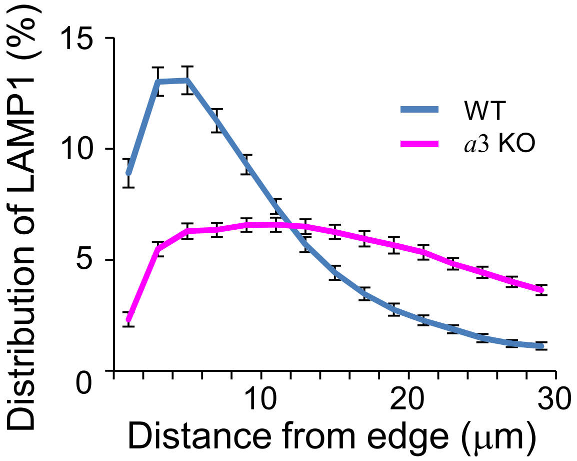

Supplementary Figure S2

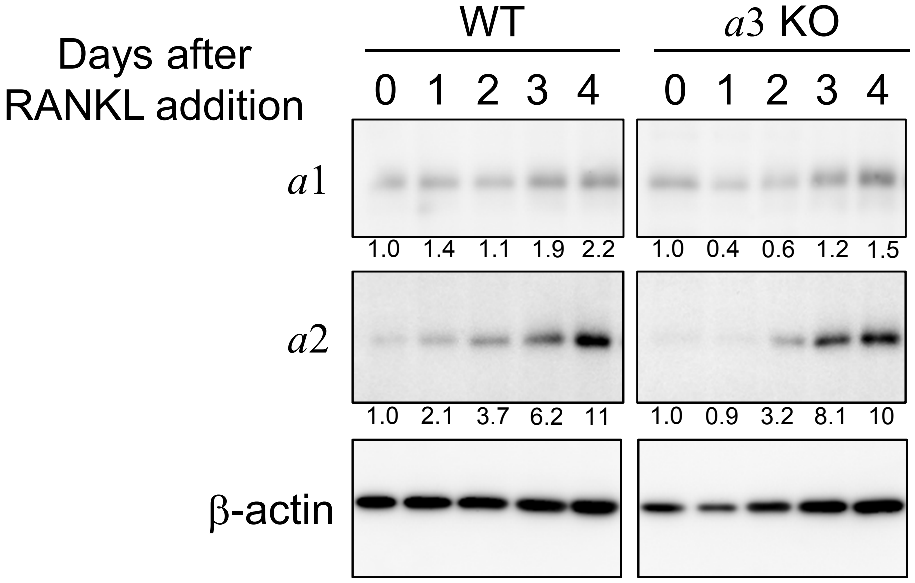

Supplementary Figure S3

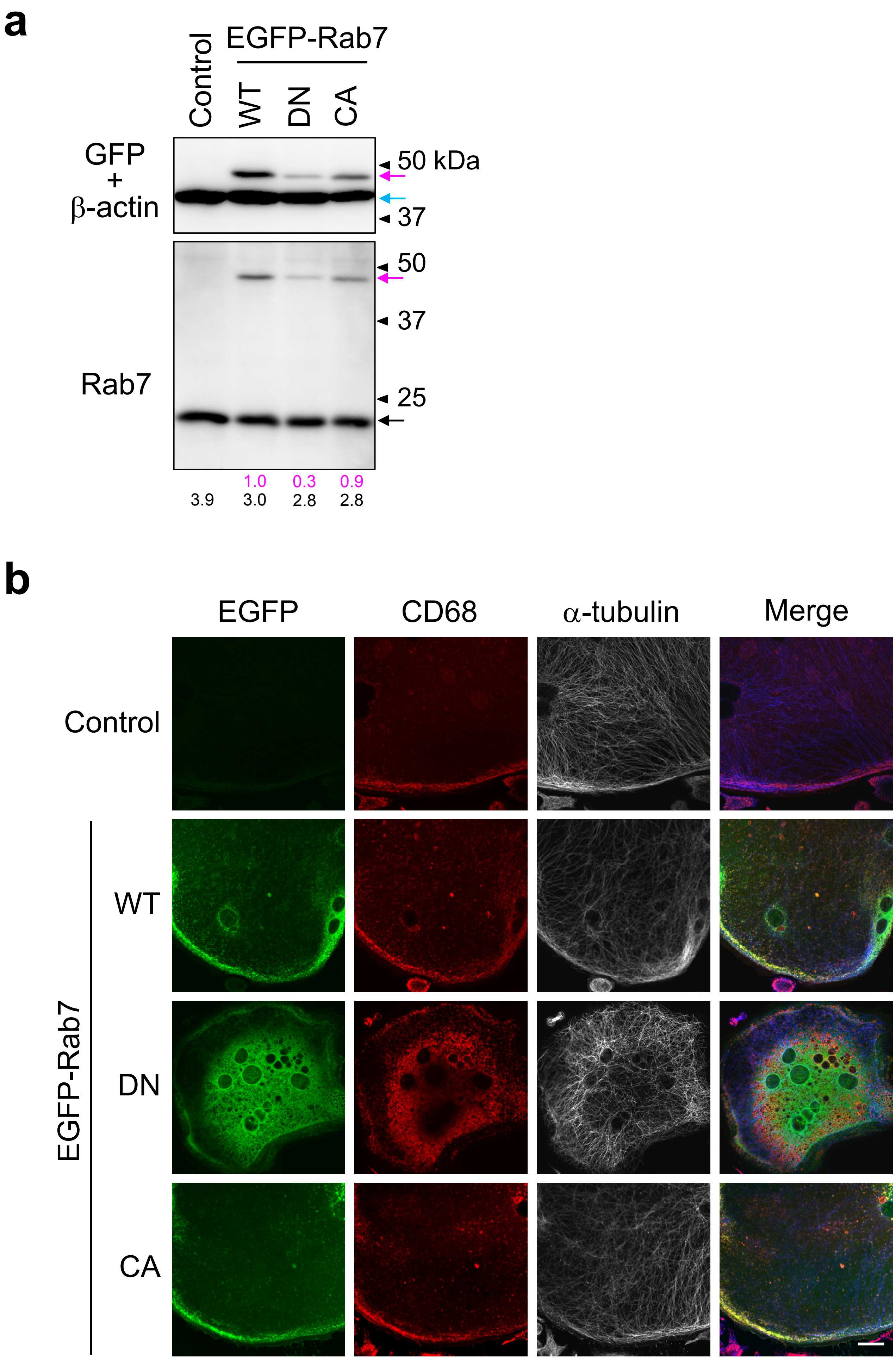

Supplementary Figure S4

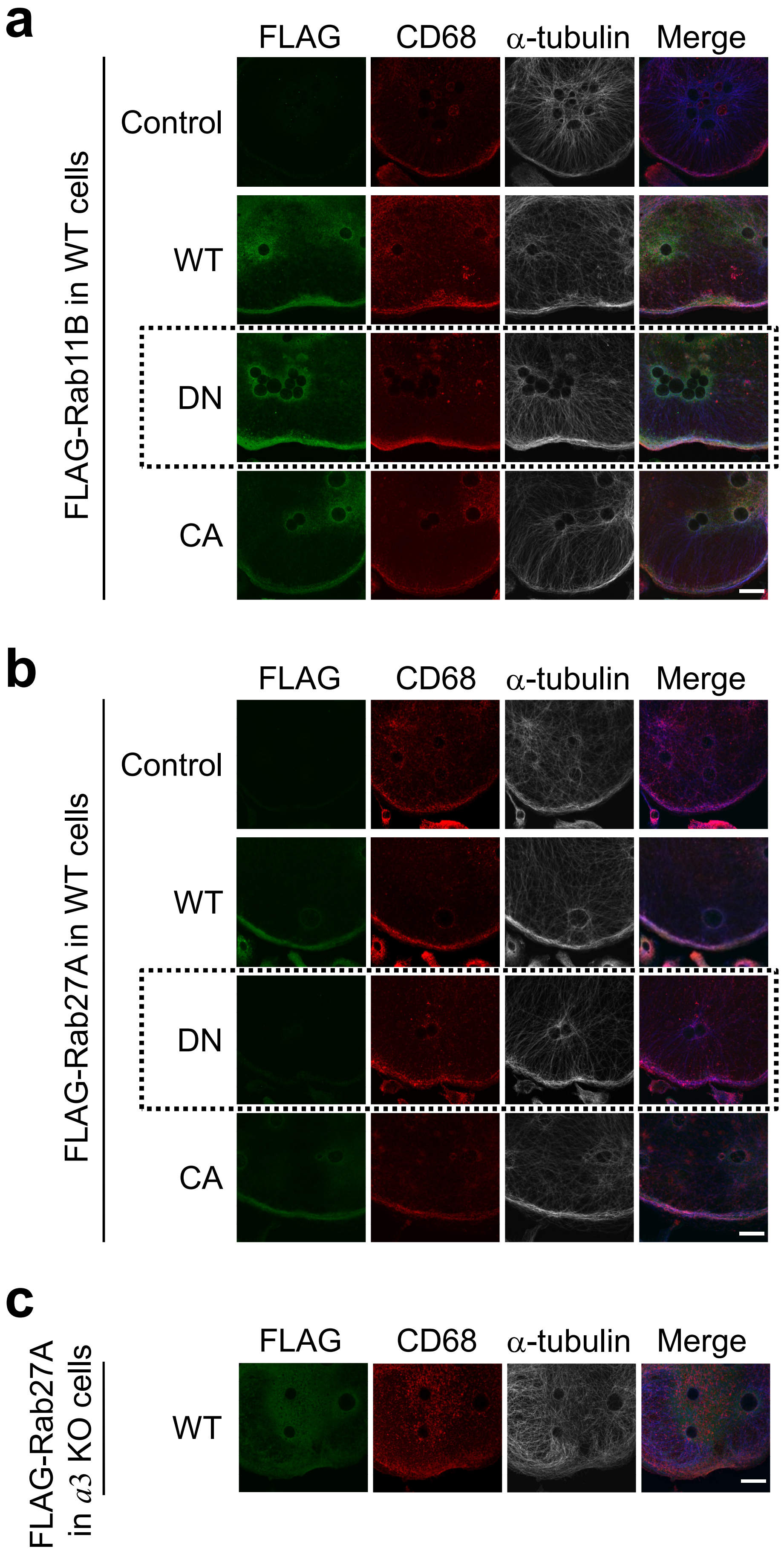

Supplementary Figure S5

Fig. 4a

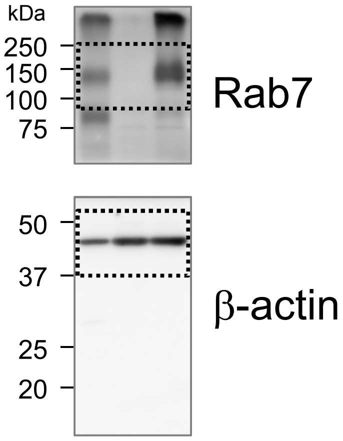

Fig. 6a

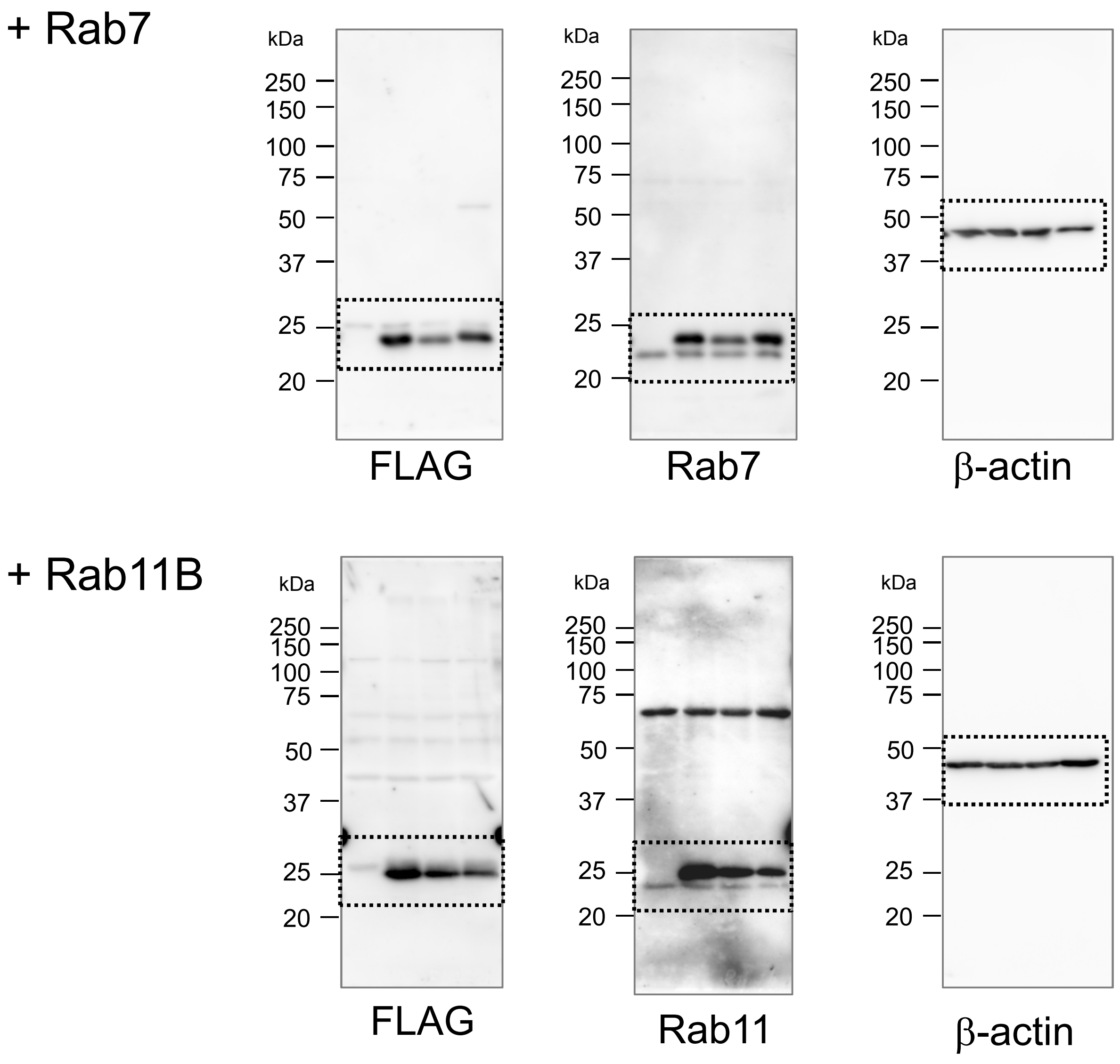

Supplementary Figure S6

Fig. 6a

+ Rab27A

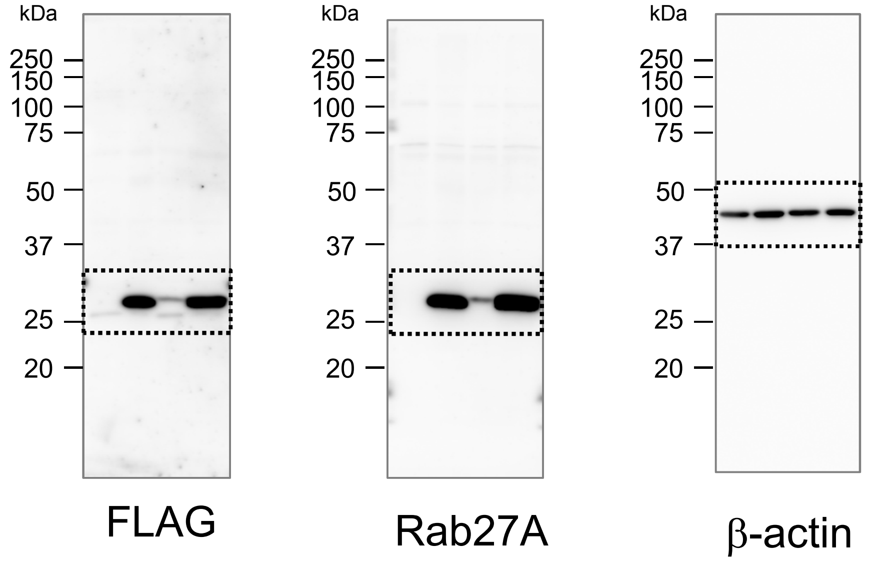

Fig. 7d

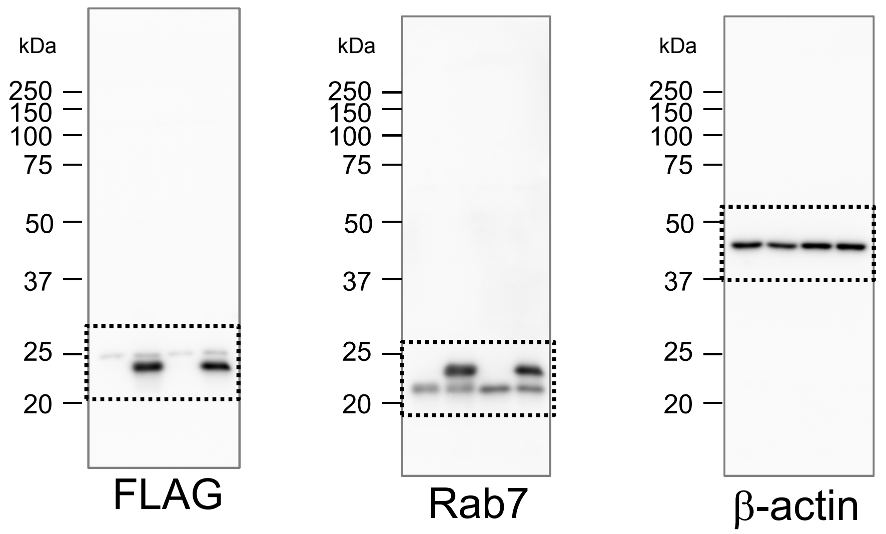

Supplementary Figure S6

Fig. 8a

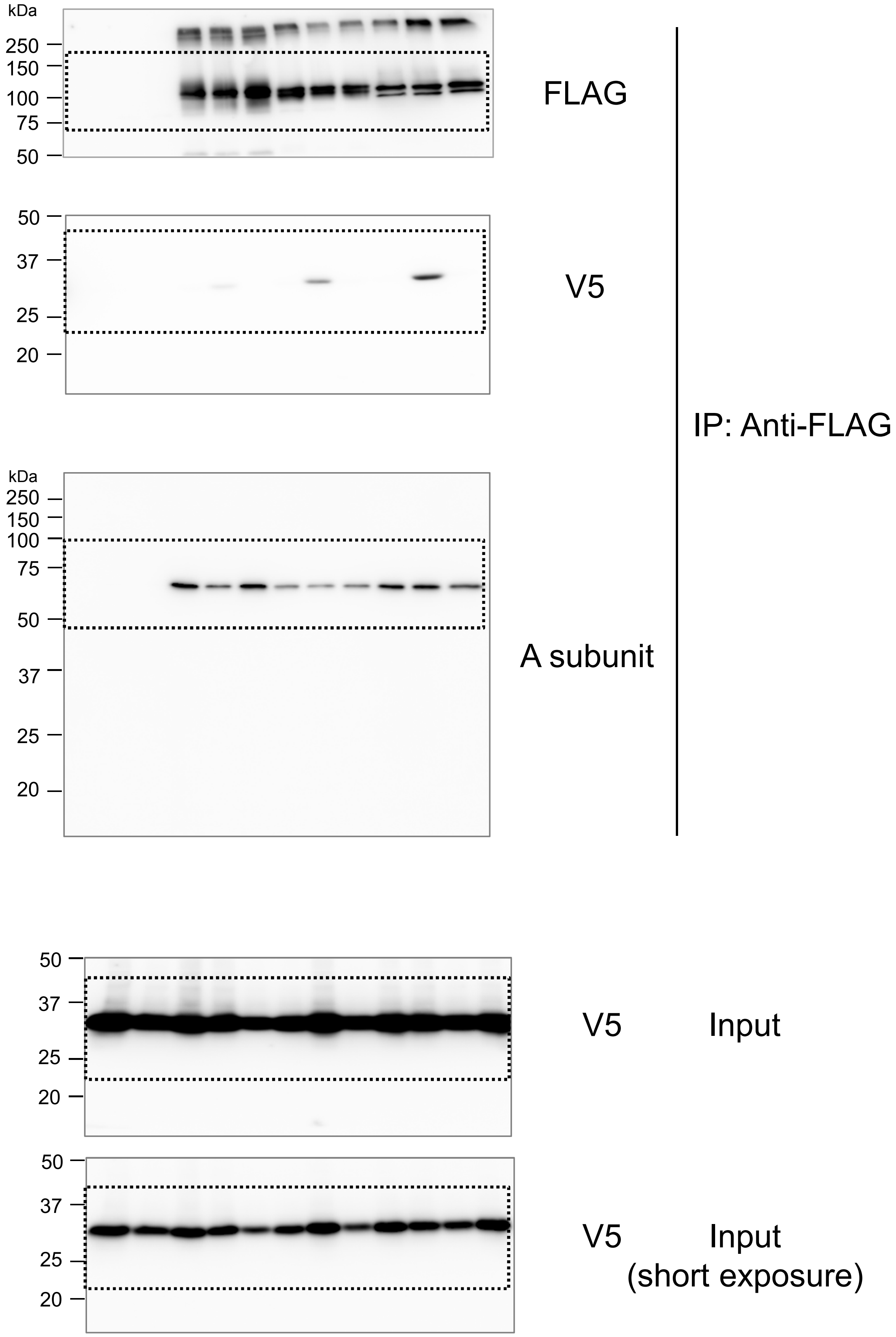

Supplementary Figure S6

Fig. 8b

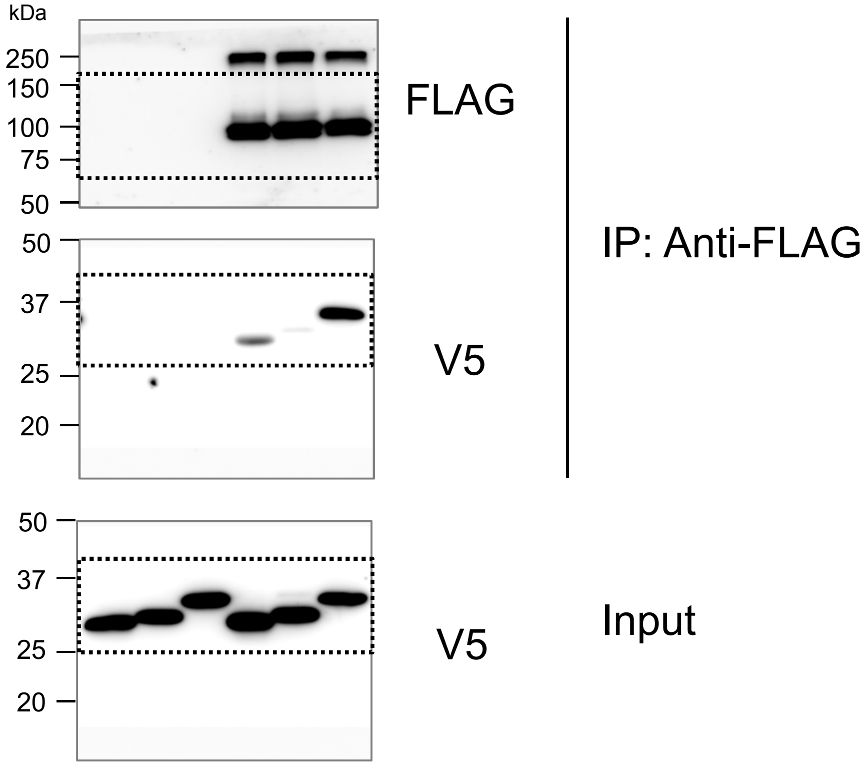

Fig. 8c

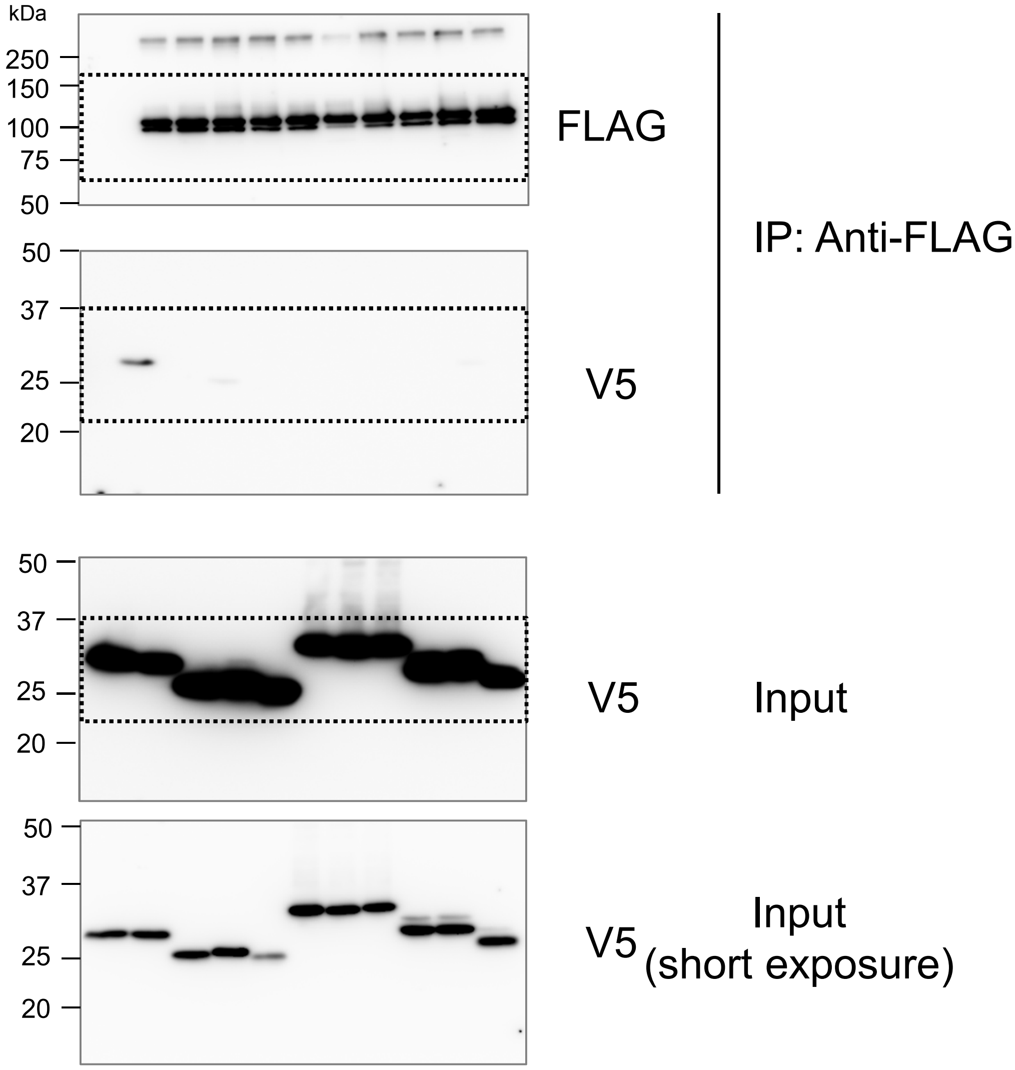

Supplementary Figure S6

Fig. S3

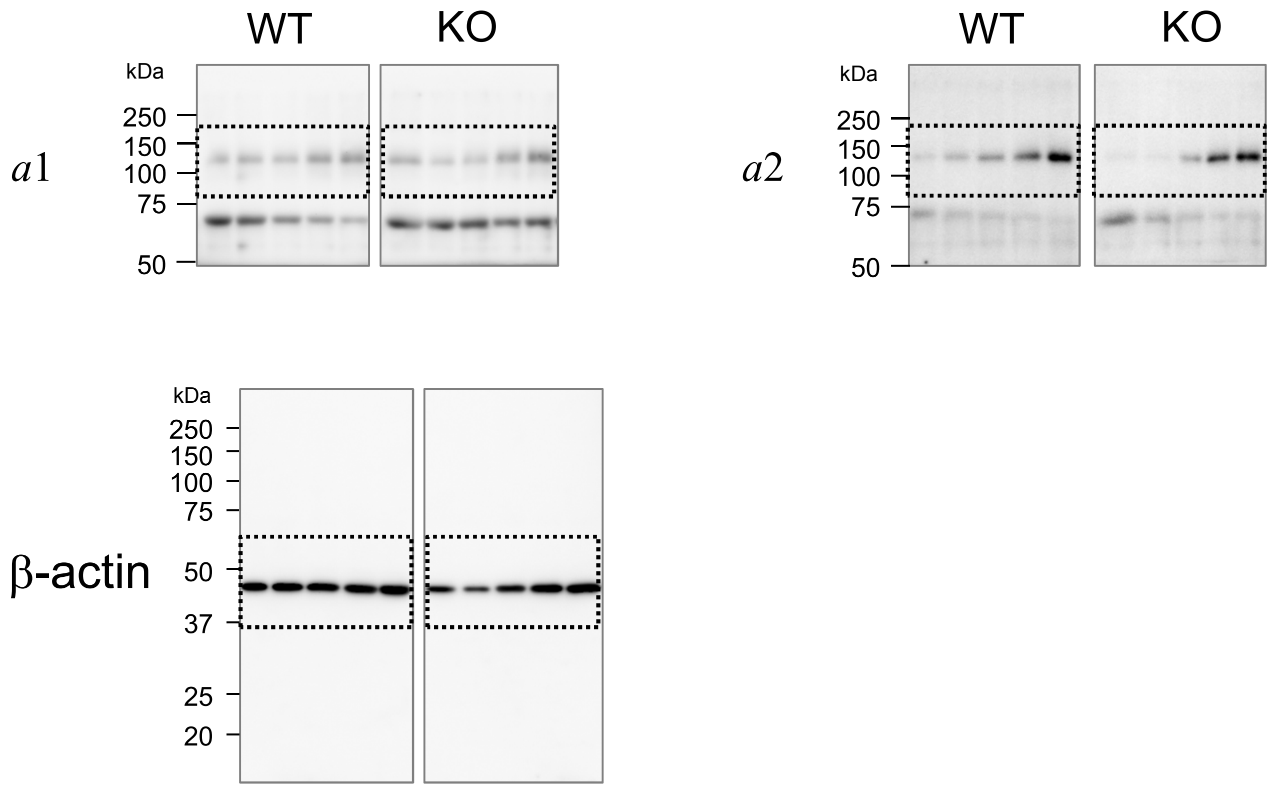

Fig. S4

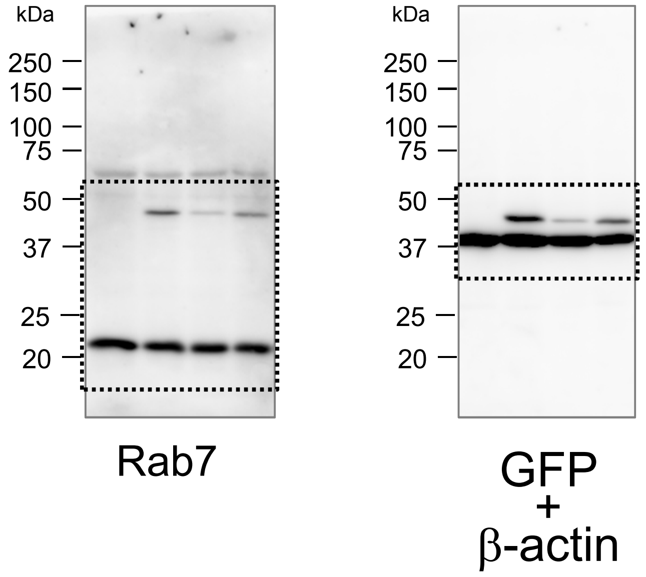

Supplementary Figure S6
